# Spatiotemporal analysis with a genetically encoded fluorescent RNA probe reveals TERRA function around telomeres

**DOI:** 10.1038/srep38910

**Published:** 2016-12-13

**Authors:** Toshimichi Yamada, Hideaki Yoshimura, Rintaro Shimada, Mitsuru Hattori, Masatoshi Eguchi, Takahiro K. Fujiwara, Akihiro Kusumi, Takeaki Ozawa

**Affiliations:** 1 Department of Chemistry, School of Science, The University of Tokyo, 7-3-1 Hongo, Bunkyo-ku, Tokyo 113-0033, Japan; 2 Institute for Integrated Cell-Material Sciences (WPI-iCeMS), Kyoto University, Kyoto 606-8507, Japan

## Abstract

Telomeric repeat-containing RNA (TERRA) controls the structure and length of telomeres through interactions with numerous telomere-binding proteins. However, little is known about the mechanism by which TERRA regulates the accessibility of the proteins to telomeres, mainly because of the lack of spatiotemporal information of TERRA and its-interacting proteins. We developed a fluorescent probe to visualize endogenous TERRA to investigate its dynamics in living cells. Single-particle fluorescence imaging revealed that TERRA accumulated in a telomere-neighboring region and trapped diffusive heterogeneous nuclear ribonucleoprotein A1 (hnRNPA1), thereby inhibiting hnRNPA1 localization to the telomere. These results suggest that TERRA regulates binding of hnRNPA1 to the telomere in a region surrounding the telomere, leading to a deeper understanding of the mechanism of TERRA function.

Telomeres, the physical ends of eukaryotic chromosomes, consist of a tandem array of 5′-TTAGGG-3′ repeats and a large set of telomere-binding proteins^1^,^2^,^3^. Because of the end replication problem, telomeres shorten with each cell division^4^,^5^. The continual loss of telomeric repeats generates dysfunctional telomeres, producing genome instability and leading to cellular senescence. Importantly, this telomere shortening is counteracted in stem or cancer cells to maintain telomere length. Another function of telomeres is the protection of chromosome ends from being recognized as DNA double-strand breaks^6^,^7^,^8^. During telomere maintenance and protection, numerous chromatin-modifying proteins and telomerase regulate the structure and length^9^,^10^,^11^. Recent advances in telomere biology have revealed that the accessibility of these telomere-regulating factors to telomeres is controlled by a long non-coding RNA, telomeric repeat-containing RNA (TERRA)^12^,^13^. TERRA is a transcription product of telomeres, consisting of a subtelomeric sequence and UUAGGG-repeats at its 3′ end^12^,^13^,^14^,^15^. Despite the fact that TERRA and chromatin-modifying factors cooperate to regulate telomere states^16^, the function of TERRA in telomere regulation remains elusive.

Biochemical and mass spectrometric studies have revealed the proteins which interact with TERRA^17^,^18^,^19^, and have proposed various models of TERRA functions for regulating the localization of its interacting proteins to telomeres^20^,^21^. Although the localization and motion of TERRA are governed by physical laws, the dynamics of TERRA in these models cannot be explained by simple Brownian motion or static confinement of TERRA at telomeres. The underlying mechanism which connects TERRA dynamics to its function remains unclear. Spatial and temporal information of TERRA, its interacting proteins, and telomeres in living cells might refine proposed mechanistic models, leading to deeper understanding of TERRA mechanisms. Here we developed a fluorescent probe to analyze the dynamics of TERRA in living cells and investigated the mechanism of TERRA function. The probe emits fluorescence upon binding to telomeric-repeats of TERRA, enabling visualization of endogenous TERRA with single-particle resolution in living cells. Using the fluorescence probe, we investigated the distributions and motions of TERRA and heterogeneous nuclear ribonucleoprotein A1 (hnRNPA1) in living cells at the single-particle level. Based on the single-particle analysis, we propose a mechanistic model by which TERRA functions as a scaffold to hold hnRNPA1 around a telomere, inhibiting the localization of hnRNPA1 to the telomere.

## Results

### Design and characterization of the TERRA probe

**Figure 1:**
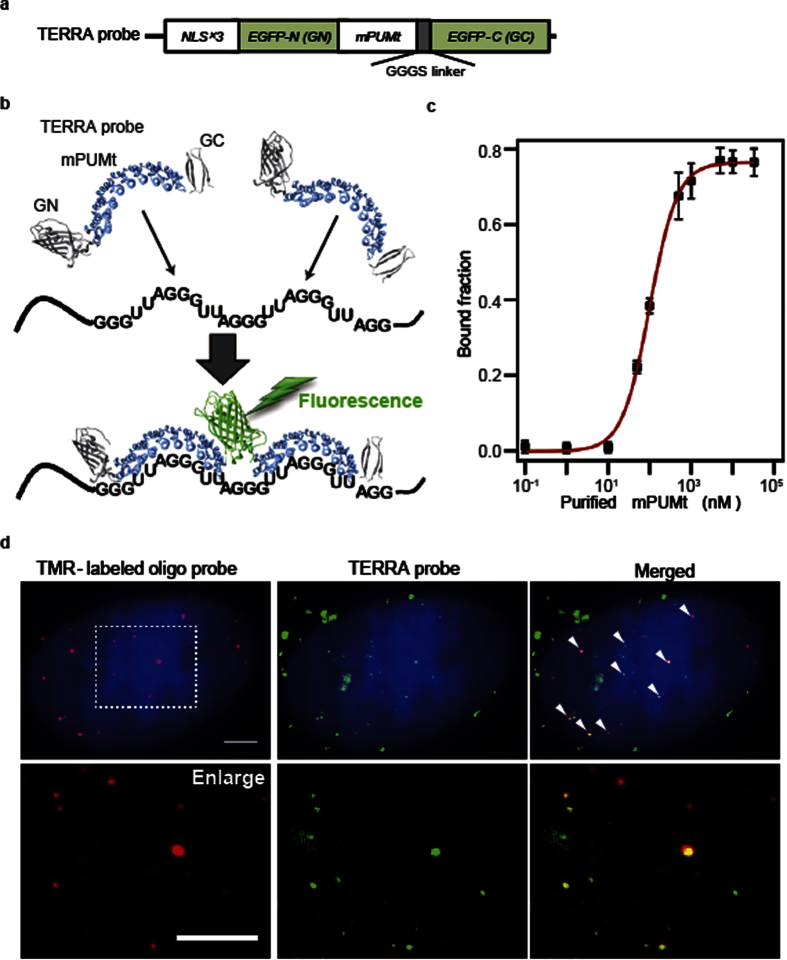
Design and characterization of the TERRA probe: (**a**) Schematic of the domain structure of the TERRA probe. The mutant PUM-HD that binds to the repetitive RNA sequence (mPUMt) is sandwiched between split fragments of EGFP (N-terminal fragment: EGFP-N, C-terminal fragment: EGFP-C). The construct contains three tandem repeats of a NLS (NLS × 3). (**b**) Mechanism used to visualize TERRA with the probe. Binding of TERRA probes to the repeated target sequence induces intermolecular reconstitution of EGFP fragments, which recovers EGFP fluorescence. (**c**) Plot of RNA-bound fraction against mPUMt concentrations. (**d**) Localization of TERRA and TERRA probes. TERRA labeled with TMR-oligonucleotide (left) and the reconstituted EGFP on the TERRA probe (right) in the same cell. The white arrowheads in the merged image (right) show probes colocalized with TERRA. Bottom panels show enlarged images of the regions indicated by white dashed lines. Scale bar, 5.0 μm.

We developed a TERRA probe consisting of a mutant of sequence-specific RNA-binding domain, Pumilio homology domain (PUM-HD), to recognize endogenous TERRA in living cells^22^,^23^,^24^. PUM-HD comprises an array of eight elements, in each of which three amino-acid residues interacts with a specific RNA base^25^,^26^. Site-directed mutations of PUM-HD alter its RNA sequence specificity^25^,^26^,^27^. To detect the UUAGGG repeat in TERRA, we designed a PUM-HD mutant (mPUMt) that binds to an eight-base sequence RNA: 5′-UUAGGGUU-3′ (Supplementary Table 1). The mPUMt sequence was inserted into a dissection site of split enhanced green fluorescent protein (EGFP) fragments (Fig. 1a). Three repeats of a nuclear localization signal (NLS) sequence were connected at the N-terminus of the EGFP to localize the probe to the nucleus (Supplementary Fig. 1a). Upon binding of the two probes to adjacent positions in a TERRA-repeat region, the EGFP fragments of the two probes are brought into proximity, thereby allowing their reconstitution, leading to the recovery of EGFP fluorescence (Fig. 1b).

We examined the affinity of mPUMt to the target RNA sequence. The dissociation constant between mPUMt and the 5′-UUAGGGUU-3′ sequence RNA was calculated as 0.10 ± 0.01 μM (Fig. 1c). The binding of mPUMt to the target RNA was not inhibited by an RNA containing a wild-type PUM-HD recognition sequence (5′-UGUAUAUA-3′, Supplementary Fig. 1b). In addition, neither single-stranded nor double-stranded DNA of the sequence TTAGGGTT hampered the interaction between mPUMt and the target RNA sequence (Supplementary Fig. 1b), thereby confirming the specificity of mPUMt for the UUAGGGUU sequence.

To confirm the EGFP reconstruction of the TERRA probe with UUAGGG repeats, we generated U2OS cells carrying transcriptionally inducible iRFP-(TTAGGG)_13_ whose transcription is regulated by doxycycline (dox). Treatment with dox for 2 hrs led to an approximately two-fold increase in the number of EGFP spots in the nucleus (Supplementary Fig. 1c,d). Moreover, when the cells were treated with dox, EGFP spots also appeared in the cytoplasm (Supplementary Fig. 1d), suggesting that the TERRA probe reconstituted EGFP on the artificially expressed UUAGGG repeats. We next investigated the probe’s ability to visualize endogenous TERRA in human osteosarcoma U2OS cells. Endogenous TERRA was hybridized with a tetramethylrhodamine (TMR)-labeled oligonucleotide (TMR-(GGGTTA)_7_) after cell fixation. A merged image revealed that 71% of EGFP fluorescent spots in the nucleus overlapped with TMR spots (Fig. 1d), thereby indicating the successful reconstitution of EGFP on the repeated RNA sequence of TERRA. To exclude the possibility of intramolecular EGFP reconstitution in the absence of TERRA, mPUMt in the TERRA probe was replaced by another PUM-HD mutant recognizing β-actin mRNA (PUM_*ACT*_)^23^. The probe containing PUM_*ACT*_ cannot reconstitute EGFP because β-actin mRNA does not possess repeated PUM_*ACT*_ recognition sequences. Although the variant localized to the nucleus, no reconstituted EGFP fluorescence was detected (Supplementary Fig. 1e). Consequently, the EGFP fragments of the TERRA probe do not reconstitute spontaneously in the absence of the target sequence. We concluded that the TERRA probe specifically labeled the UUAGGG-repeated sequence by EGFP reconstitution in the cells.

The effects of the TERRA probe on telomere structure were examined by the quantitative telomere FISH method on single telomeres from metaphase spreads. The expression of the TERRA probe did not cause any significant changes in average telomere length, which were determined by calculating fluorescence intensity of individual telomeres (Supplementary Fig. 2a). Moreover, we investigated the ratio of telomere aberration by calculating the number of a telomere free end (TFE) which was defined as the chromosome end without telomere (Supplementary Fig. 2b). The percentage of TFE from the cells with the TERRA probe was 16.8% (n = 184). The value was comparable to that without the probe (12.0%, n = 101, Supplementary Fig. 2c). These results indicated that the TERRA probe does not inhibit the ability of TERRA to maintain the telomere length and structure.

### Live cell imaging of TERRA

**Figure 2:**
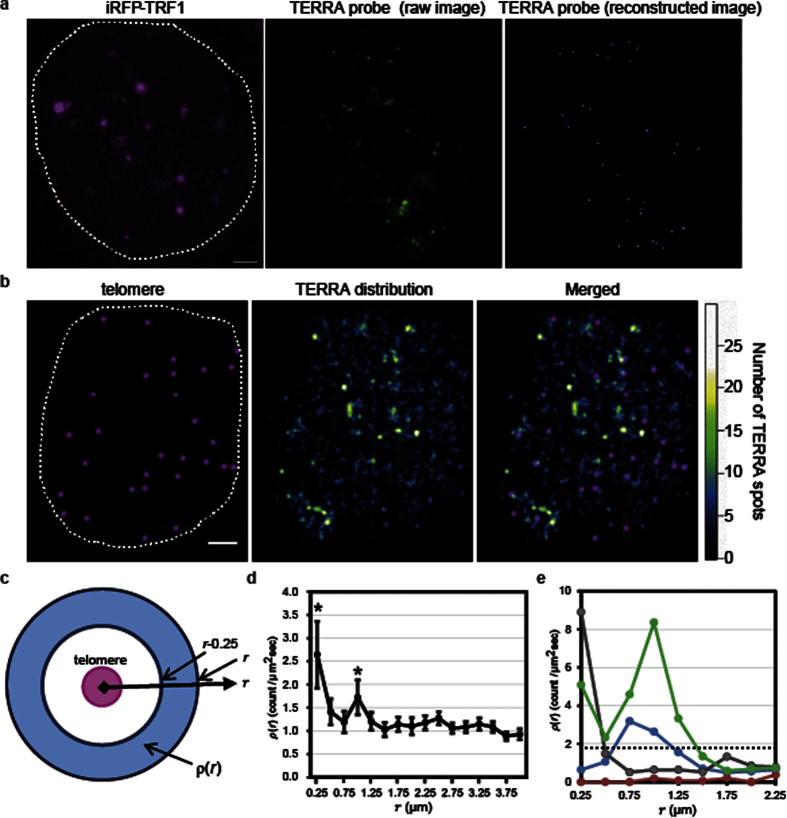
Analysis of TERRA distribution. (**a**) Live-cell imaging of TERRA molecules. Fluorescence images were obtained using a representative U2OS cell expressing iRFP-TRF1 (left), TERRA probe (middle), and a single-particle reconstruction of TERRA from the middle image (right, movie S1 and S2). The white broken line in the left panel outlines the nucleus. Each Gaussian spot in the right image is reconstructed as a spot with 200 nm diameter. Scale bar, 2.0 μm. (**b**) Overlaid images of fluorescent spots representing telomeres (left) and TERRA (right) in movie S1. Time-integrated images were constructed to show the spatial distributions of telomeres and TERRA foci. The TERRA density is shown in pseudo-color. Scale bars, 4.0 μm. (**c**) Schematic of the radial distribution function, ρ(*r*). ρ(*r*) is the number of TERRA spots divided by the observation time and area within the region between *r* − 0.25 and *r* (μm) (blue) from the center of a reference telomere (magenta). (**d**) Averaged ρ(*r*) of the TERRA spots for all the telomeres in the cell shown in Fig. 2A (±s.e.m., n = 26). ρ(*r* = 0.25 μm) and ρ(*r* = 1.00 μm) exceed ρ(*r* > 3.00 μm) (Student’s *t*-test, *p* < 0.05). (**e**) Plots of ρ(*r*) against *r* for the four individual telomeres shown in Fig. 2b. The dotted line represents the threshold for the peak (ρ(*r*) = 1.9).

Using the TERRA probe, we next investigated localization of TERRA and telomeres in living U2OS cells. The TERRA probe was expressed in cells in which telomeric DNA was labeled with stably expressed near-infrared fluorescent protein (iRFP)-TRF1^28^,^29^. TERRA was visualized as individual fluorescent particles (Fig. 2a and Video 1), whereas the iRFP-TRF1 localized extensively to telomeres and was observed as punctate fluorescence spots in the nucleus (Video 2). We next evaluated the number of EGFP molecules in the single fluorescent spots by estimating time-course intensity profiles of the fluorescent spots. A fluorescent spot showed fluctuation in its fluorescence intensity and photobleached in a single-step fashion (Supplementary Fig. 3a), demonstrating that the spot corresponds to a single EGFP. The histogram of the fluorescence intensity distribution of all the detected spots was fitted with a single Gaussian curve (Supplementary Fig. 3b), indicating that the analyzed fluorescent spots consisted of the same number of the reconstituted EGFPs. Taken these two results together, we concluded that the fluorescent spots represented single EGFPs. Because of the weak fluorescence of the TERRA probe, background fluorescence hampered precise analysis of the TERRA distribution. To eliminate the background fluorescence, we performed position detection of individual EGFP spots using cross-correlation^30^. The spot-detectable thickness was ±200 nm from the center of the focus plane, as confirmed by the observation of fluorescent beads with different vertical positions (Supplementary Fig. 4a and b). Then, the EGFP spots from the raw data were fitted with Gaussian spots of 200 nm diameter, corresponding to the full width at half maximum (FWHM) of the EGFP spots in the raw image (Fig. 2a). Similarly, Gaussian spots with diameters of 500 nm were shown at the positions of iRFP-TRF1 (Video 3). Time-integration of the Gaussian spots showed that TERRA formed foci in specific regions in the nucleus (Fig. 2b). The ratio of telomeres colocalizing with TERRA foci was 8.9%. That result is consistent with previous ones obtained by fluorescence *in situ* hybridization of TERRA^29^,^31^. Intriguingly, several TERRA foci were 1.0–2.0 μm apart from the center of telomeres.

To determine whether the foci represent the characteristic of TERRA, we visualized temperature-dependent alteration of TERRA foci using the TERRA probe. A previous study showed that thermal stress on MEF cells induces reversible increment in the number of TERRA foci^13^. To demonstrate the ability of the probe to visualize the thermal stress-induced increasing in the number of TERRA foci, we prepared three cell samples cultured in different temperature conditions; one was the condition in which the cells were cultured at 37 °C, the second was that of the cells heat-shocked at 42 °C for 1 hr before the observation, and the last is the condition where the temperature was restored to be 37 °C after the 42 °C heat shock. We obtained movies of the fluorescence observations on the three samples and integrated all the frames to elicit TERRA foci (Supplementary Fig. 5a). The cells with a heat shock at 42 °C for 1 hour resulted in an increase in the number of TERRA foci in comparison to the sample cultured in constant temperature at 37 °C (Supplementary Fig. 5b). In the cells with recovering the temperature back to be 37 °C after the heat shock treatment, the number of TERRA foci decreased to the comparable level to that in the cells without the heat shock (Supplementary Fig. 5b). These results are consistent with the previous reports based on FISH^13^, demonstrating that the TERRA probe enable to visualize the alteration of the number of TERRA foci in living cells.

### Quantification of spatial distribution and motion of TERRA

To quantify the distance between a telomere and surrounding TERRA foci, we introduced a radial distribution function (ρ(*r*)) that evaluates the TERRA density in a region at a specific distance from the telomere center (Fig. 2c). We changed the radius (*r*) by 0.25*-*μm increments. Upon averaging ρ(*r*) for all telomeres in the images, two peaks of high TERRA density were detected at distances of 0.25 and 1.00 μm (*p *< 0.05, Fig. 2d). To confirm the specificity of the peak at *r* = 1.00 μm, we calculated ρ(*r*) from arbitrary positions outside of the telomeres. Under these conditions, a peak was detected only at *r* = 0.25 μm (Supplementary Fig. 6a). In addition, the ρ(*r*) of hnRNPA1 was calculated, revealing no ρ(*r*) peak for hnRNPA1 (Supplementary Fig. 6b). These data suggest that the peak at *r* = 1.00 μm is characteristic of the TERRA distribution around a telomere. The ρ(*r*) values calculated for individual telomeres showed variation in distribution patterns of TERRA around telomeres (Fig. 2e). We defined local maxima of ρ(*r*) as greater than twice ρ(*r* > 3.50 μm). Based on this definition, the ρ(*r*) was categorized into four patterns: no peak (41%); a single peak on a telomere (*r* < 0.50 μm, 20%); a single peak near telomere (0.75 < *r* < 2.00 μm, 29%); and two peaks on and near a telomere (10%, Fig. 2e, Supplementary Fig. 6c). These results indicate that 39% of telomeres were accompanied by TERRA foci within 1–2 μm.

**Figure 3:**
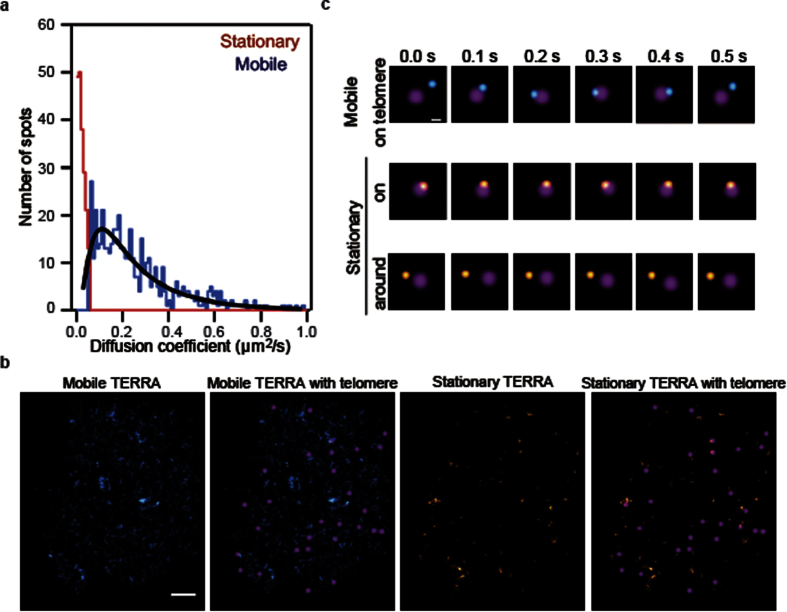
Analysis of TERRA motion: (**a**) Histogram of the diffusion coefficient distribution of stationary TERRA (red, *n* = 200) and mobile TERRA (blue, *n* = 502) with a minimum track length of 7 frames (700 ms). Distribution of the diffusion coefficients of mobile TERRA was fitted with a lognormal function (black line). (**b**) Spatial distribution of TERRA in mobile and stationary phases. Mobile TERRA spots with D > 5.53 × 10^−2^ μm^2^/s are shown in cyan. The spots in the stationary phase are shown in orange. The images are overlaid with the localization of telomeres (magenta). Scale bar, 2.0 μm. (**c**) Sequential images of stationary and mobile TERRA on or near a telomere. Scale bars, 0.5 μm.

We next used the TERRA probe to analyze the dynamics of individual TERRA molecules in living cells. The motion of each TERRA spot was characterized by its diffusion coefficient (*D*_TERRA_). The *D*_TERRA_ values for individual TERRA molecules were widely distributed, ranging from 5.90 × 10^−4^ to 1.28 μm^2^/s, implying the existence of multiple modes of TERRA motion. To define a stationary mode, we estimated the apparent diffusion coefficients (*D*_fix_) of TERRA with fixed cells. Considering that the diffusion coefficient calculated from trajectories with limited steps shows wide distribution and is fitted well with a lognormal function rather than a single Gaussian function^32^,^33^, we performed curve fitting of the diffusion coefficient distribution with single lognormal functions. The distribution of *D*_fix_ was fitted with a lognormal function for which the 99% confidence limit was 5.53 × 10^−2^ μm^2^/s (Supplementary Fig. 7a). Then, we defined the motion of TERRA spots in living cells with *D*_TERRA_ values lower than the 99% confidence limit as the stationary mode (Video 4). From this classification, we found that 29% of the total TERRA was in the stationary mode, whereas the remainder was in a mobile mode. The *D*_TERRA_ distribution of the mobile TERRA was fitted with a lognormal distribution (mean; 0.15 μm^2^/s, variance; 0.02 μm^2^/s, Fig. 3a). The mean *D*_TERRA_ was of the same order of magnitude as that previously reported on poly(A) RNA in living cells^34^,^35^, suggesting that TERRA in the mobile mode diffuses freely in a manner similar to that of other RNAs in the nucleus. Notably, two modes of TERRA were present at telomeres (*r* < 0.50 μm): a stationary mode (38%) and a mobile mode (62%, Fig. 3b). Numerous stationary TERRA molecules were localized to telomeres during the observation period (Fig. 3c). In contrast, mobile TERRA molecules colocalize transiently with a telomere for 0.1–0.3 s and were largely diffuse in the nucleoplasm (Fig. 3c). Moreover, the ratio of stationary TERRA in the telomere-neighboring region (0.75 < *r* < 2.00 μm) was 42%, which was higher than that in the nucleoplasm (29%, *p* < 0.001), suggesting that the motions of TERRA are confined in the telomere-neighboring region.

### Dynamics of TERRA-hnRNPA1 complex in living cells

Most of the models of TERRA functions are based on the hypothesis that TERRA works as a scaffold in telomere^19^,^21^. The existence of stationary TERRA around telomeres suggests another mechanism of the TERRA function. Recently, it was suggested that TERRA inhibits the localization of hnRNPA1 to telomeres, which cannot be explained using the scaffold model^20^. To assess the possibility of inhibitory activity of TERRA on hnRNPA1, we examined the dynamics of TERRA, hnRNPA1, and telomeres in living cells by single-particle tracking. Expression plasmids for the TERRA probe and SNAPtag-fused hnRNPA1 were transfected into cells stably expressing iRFP-TRF1. After staining the expressed SNAP-hnRNPA1 with TMR, we performed simultaneous observation of the three factors by their respective fluorescence signals. We classified the motions of hnRNPA1 into stationary or mobile modes. This classification revealed that only 29% of the hnRNPA1 on telomeres was stationary and that the remainder was mobile (*r* < 0.50 μm, Supplementary Fig. 7c and d), indicating that localization of hnRNPA1 to telomeres is transient.

**Figure 4:**
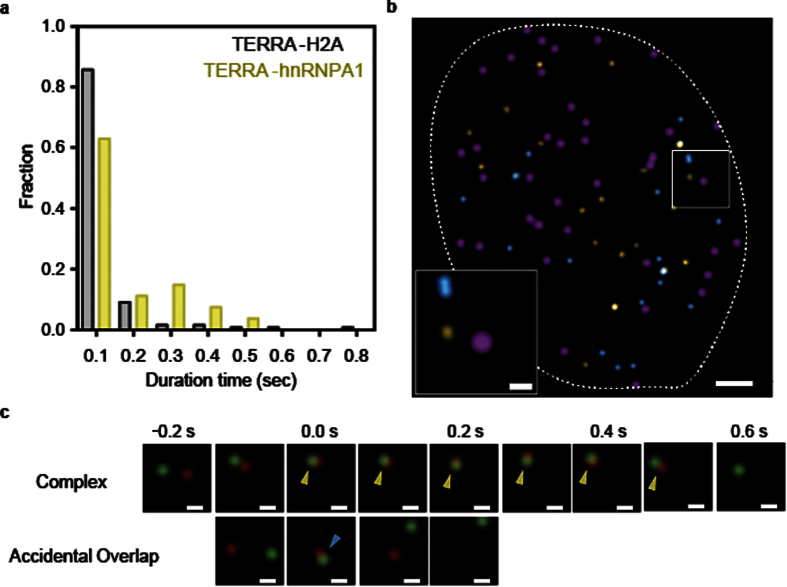
Definition of TERRA-hnRNPA1 complexes in living cells. (**a**) Distributions of the duration of TERRA colocalization with hnRNPA1 (yellow) and H2A (gray). In all, 130 colocalization events were analyzed for the respective proteins. (**b**) Spatial distribution of the colocalization between TERRA and hnRNPA1 in the nucleus. TERRA-hnRNAP1 complexes are shown as yellow spots. Accidental overlaps are shown as cyan spots. Telomeres are represented by magenta spots. Scale bars, 2.0 μm and 0.5 μm (inset). (**c**) Sequential images of the colocalization of a TERRA spot (green) and an hnRNPA1 spot (red). Overlapping events that exceeded 0.2 s (yellow arrowheads) show TERRA-hnRNPA1 complex formation. Overlap sustained only for 0.1 s (cyan arrowhead) was regarded as an accidental overlap. Scale bars, 0.1 μm.

Next, we calculated the distance between TERRA and hnRNPA1 to detect TERRA-hnRNPA1 complexes in living cells. Colocalization of TERRA with hnRNPA1 was defined according to their localization precisions (32 nm for TERRA and 28 nm for hnRNPA1, Supplementary Fig. 8) and the overlay accuracy (30 nm). To ascertain whether colocalization resulted from an accidental overlap or complex formation, we investigated the durations of individual colocalization events. To define the accidental overlap of two spots, we evaluated the colocalization of TERRA with H2A, a protein that does not interact with TERRA^36^. The majority of the colocalization between TERRA and H2A, which occurred through the accidental overlap, lasts only 0.2 s (Fig. 4a). For the colocalization of TERRA with hnRNPA1, the fraction of events in which the colocalization duration exceeded 0.2 s was 26% of the total number of colocalization events, which was remarkably higher than that for colocalization with H2A (6.1%). Therefore, the colocalizations of TERRA with hnRNPA1 exceeding 0.2 s were defined as TERRA-hnRNPA1 complexes (Fig. 4b and c, Video 5).

**Figure 5:**
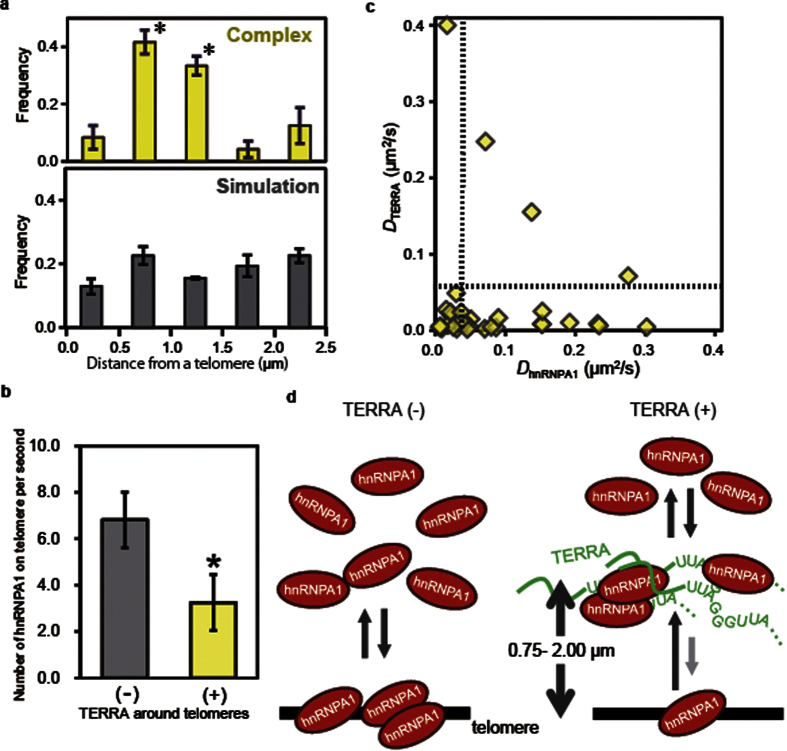
Relation between TERRA function and spatiotemporal dynamics of TERRA-hnRNPA1 complexes. (**a**) Fraction of colocalized regions shown as a function of the distance from a telomere: yellow, TERRA-hnRNPA1 complex formation (* indicates *p* < 0.05, Student’s *t*-test); gray, simulated colocalization of TERRA with a freely diffusing molecule. (**b**) Correlation between hnRNPA1 localization to a telomere and TERRA accumulation around the telomere. The hnRNPA1 spots on telomeres were counted. During the observation, telomeres were categorized into two groups based on TERRA localization: TERRA localized to a telomere-neighboring area (TERRA around telomeres: +, *n* = 13) and no TERRA located around a telomere (TERRA around telomeres: −, *n* = 14). When TERRA was absent around a telomere, the number of hnRNPA1 occurrences was 6.8 spots/s, whereas this value was reduced by half when TERRA was present in the telomere-neighboring area (average: 3.2 spots/s, *p* < 0.05). (**c**) Scatter plot of the diffusion coefficients of TERRA and hnRNPA1 including a period of complex formation within the trajectory. Broken lines show thresholds between the stationary and mobile phases. (**d**) Model showing how TERRA regulates the localization of hnRNPA1 on a telomere. When TERRA is absent around a telomere (shown as a black bar), the binding of hnRNPA1 to the telomere is at equilibrium (left). When TERRA localizes to the telomere-neighboring region (0.75–2.00 μm from the telomere center), the locally accumulated TERRA captures hnRNPA1, shifting its equilibrium toward the telomere-unbound form (right).

To analyze the spatial distribution of TERRA-hnRNPA1 complexes, the percentage of complexes was shown against the distance from a telomere (Fig. 5a). The plot exhibited a local maximum in the telomere-neighboring region (0.5 < *r* < 1.5 μm). We also evaluated the colocalization of TERRA and freely diffusing spots generated by computer simulation. The spatial distribution of the colocalization between TERRA and the freely diffusing spots was homogeneous around telomeres (Fig. 5a). These results indicate that the TERRA-hnRNPA1 complex forms intensively in the telomere-neighboring region. Although both TERRA and hnRNPA1 localized to telomeres (Fig. 2d, Supplementary Fig. 7d), the frequency of complex formation on a telomere was less than 0.1 (Fig. 5a), which was comparable to that in the nucleoplasm. These results suggest that the TERRA in telomere-neighboring areas, rather than that at a telomere, is responsible for the delocalization of hnRNPA1. To test this hypothesis further, we investigated whether hnRNPA1 localization to a telomere depends on the presence of TERRA in the telomere-neighboring region. In the presence of TERRA in a telomere-neighboring region, the number of hnRNPA1 spots on the telomere was lower by half (*p* < 0.05, Fig. 5b) than that on a telomere without TERRA around it. Moreover, we explored the ability of TERRA to inhibit hnRNPA1 localization to telomere by depleting endogenous TERRA. Analysis of real-time PCR revealed that two different siRNAs (siTERRA-1 and siTERRA-2) made the TERRA level depleted to ~40% of control levels (Supplementary Fig. 9a)^37^. The effect of TERRA depletion on hnRNPA1 diffusion was examined by calculating the diffusion coefficients (Supplementary Fig. 9b). We found no significant difference in the diffusion coefficients between control and TEERA knock down (KD, Supplementary Fig. 9c), suggesting that TERRA has no effect on free-diffusing hnRNPA1 in nucleoplasm. Importantly, the number of hnRNPA1 spots on telomeres in the TERRA KD cells was twice larger than that on a telomere with TERRA around it in the control cells, and was comparable to that on a telomere without TERRA around the telomere (Supplementary Fig. 9d). Consequently, we concluded that hnRNPA1 localization to telomeres is prevented when TERRA accumulates around the telomere and forms complexes with hnRNPA1.

To address the mechanism by which the TERRA near a telomere inhibits the localization of hnRNPA1 at the telomere, we analyzed the diffusion coefficients of TERRA and hnRNPA1 that form complexes mutually. Based on the analysis, only 24% of hnRNPA1 was categorized in the stationary mode, in contrast to 88% of TERRA in stationary mode (Fig. 5c). This result suggests that complex formation occurs primarily between stationary TERRA and mobile hnRNPA1 in telomere-neighboring regions.

## Discussion

We developed a fluorescent probe for endogenous TERRA to visualize its localization and dynamics in living cells. The TERRA probe bound specifically to 5′-UUAGGG-3′ repeats in TERRA and reconstituted EGFP. Although multiple EGFP can be, in principle, reconstituted by the TERRA probes at the single TERRA molecule, all of the detected spots included a single EGFP molecule in the single spots. There are several possible reasons for this: First, the amount of the TERRA probe was kept low by 4 hours incubation after the transfection for live cell imaging, which was shorter than manufacturer’s protocol (24–48 hours). Second, PUM-HD interacts with stretched or flexible RNA sequence but cannot recognize higher structure of RNA^27^: 5′-UUAGGG-3′ repeat regions of TERRA form stable parallel-stranded G-quadruplex structure which will inhibit binding of PUM-HD to TERRA^38^,^39^. Using the TERRA probe, we analyzed the density of TERRA around each telomere quantitatively. By calculating the radial distribution function of TERRA around a telomere, the TERRA distribution was categorized into four patterns: absence (40%); at the telomere (20%); near the telomere (0.75–2.00 μm distant from the telomere, 30%); both at and near the telomere (10%). Moreover, analysis of TERRA motion revealed that the population of the stationary TERRA near a telomere was 42%, which is similar to that at a telomere (38%) and higher than that in nucleoplasm (30%). These results suggest that TERRA significantly accumulated in the region apart (approx. 1.0 μm) from a telomere. It has been proposed that telomere-bound TERRA acts as a molecular scaffold to promote recruitment of telomere-binding proteins to the telomere^19^,^40^,^41^. Therefore, we hypothesize that TERRA provides scaffolds for telomere-binding proteins in the telomere-neighboring region to regulate the accessibility of the proteins to the telomeres.

We tested the hypothesis by examining whether TERRA in the telomere-neighboring region regulates the localization of hnRNPA1 to the telomere^20^,^42^. First, we quantified the density of hnRNPA1 around a telomere, which revealed that hnRNPA1 showed homogeneous distribution around a telomere in contrast to the case of TERRA. Moreover, analysis of hnRNPA1 motion revealed that a large fraction of hnRNPA1 at telomeres was in mobile mode (71%). The mobile hnRNPA1 molecules were confined transiently at telomeres for only 0.1–0.3 s. These results suggest that binding of hnRNPA1 to a telomere is not static, but rather is in a certain equilibrium state. Intriguingly, the percentage of hnRNPA1 at a telomere decreased with the existence of TERRA in the telomere-neighboring region. These results suggest that TERRA in the telomere-neighboring region inhibits hnRNPA1 localization at the telomere.

We further investigated the mechanism underlying the negative correlation between TERRA near a telomere and hnRNPA1 at the telomere by analyzing the formation of TERRA-hnRNPA1 complexes. Notably, the complex formation occurred specifically in the telomere-neighboring region. This specific formation of TERRA-hnRNPA1 complex raised the possibility that TERRA near the telomere traps hnRNPA1 to shift the equilibrium toward an hnRNPA1-unbound state of the telomere. This idea was supported by results of analysis of TERRA and hnRNPA1 motions in the complexes. Analysis of the diffusion coefficient revealed that 88% of TERRA which formed the complex was in stationary mode, whereas only 24% of hnRNPA1 was in stationary mode, indicating that stationary TERRA traps the diffusing hnRNPA1 in the telomere-neighboring region.

Based on these results, we propose that these characteristic distributions and dynamics of TERRA correlate with its function: TERRA controls the on/off binding of hnRNPA1 to a telomere by acting as a scaffold for hnRNPA1 near the telomere (Fig. 5d). In this model, the binding of hnRNPA1 to a telomere occurs at certain equilibrium in the absence of TERRA. When TERRA localizes to the telomere-neighboring region, the locally accumulated TERRA captures diffusive hnRNPA1, shifting the equilibrium toward the hnRNPA1-unbound state of telomere. Biochemical studies showed that incubation of hnRNPA1-coated telomere DNA with TERRA led to isolation of naked telomere DNA, suggesting that the presence of TERRA at telomeres promote the dissociation of hnRNPA1 from the telomere^20^,^42^. Based on this result, they proposed a model by which hnRNPA1 shuttles between a telomere and TERRA dynamically when the concentration of TERRA is high. In this study, we refined this model further by single-particle analysis of TERRA and hnRNPA1: hnRNPA1 shuttles between a telomere and nucleoplasm even without the existence of TERRA; TERRA localized in a telomere-neighboring region, rather than at a telomere, interacts with hnRNPA1 and contributes to hnRNPA1 dissociation from the telomere.

This study demonstrated live-cell imaging of TERRA at the single-particle level. By analyzing the TERRA and hnRNPA1 dynamics, we connected the distribution and motion of TERRA with its function. This study demonstrates the potential of live-cell imaging of RNAs in studying RNA functions of interest. Single-particle analysis reveals the relation between RNA dynamics and its function, establishing the mechanistic model based on the spatiotemporal information in living cells. The probe developed in this study can be broadly applicable for various RNAs because of its flexibility of PUM-HD recognition. The PUM-based probe with a single-particle tracking technique is a powerful tool for investigating the mechanistic insights into such RNA functions. It can reveal the significance of RNA dynamics in living cells.

## Methods

### Plasmid construction

All cDNAs were cloned using pBlueScript (Stratagene, CA). The cDNAs encoding the RNA-binding domain of human PUMILIO1 (PUM-HD) (828–1176 amino acids) and the N-terminal and C-terminal EGFP fragments (GN; 1–157 amino acids, GC; 158–238 amino acids) were generated by polymerase chain reaction (PCR). To generate mPUMt, site-directed amino acid substitutions of PUM-HD (S863N, C935S, Q939E, 971 N, Q975S, C1007S, Q1031E, N1043C, S1079N, and E1083Q) were done using a mutagenic PCR technique (Table S1). The cDNAs GN and GC were ligated to mPUMt to yield GN-mPUMt-GC. Three tandem repeats of the NLS sequence DPKKKRKV were connected with the N-terminus of the GN portion (Fig. S1). The SNAPf coding region of the hnRNPA1-SNAPf expression vector was prepared with PCR using the pSNAPf-vector (NEB, NA) as the PCR template and primers to add a flexible GGSGGS linker on the N-terminus. The hnRNPA1 cDNA was obtained from Addgene (pET9d-hnRNPA1). hnRNPA1 was fused to the N-terminus of SNAPf. The TRF1 cDNA was generated by PCR and was conjugated to the N-terminus of iRFP^43^. The (TTAGGG)_13_ oligo DNA was purchased from Invitrogen Corp. and was conjugated to the C-terminus of iRFP. NLS-GN-mPUMt-GC and hnRNPA1-SNAPf were subcloned into the mammalian expression vector pcDNA3.1 (+) (Invitrogen Corp., CA). Then iRFP-TRF1 was inserted into the retrovirus expression vector pCLNCX. iRFP-(TTAGGG)_13_ was inserted into a Tet-on inducible vectors, which is an EBV-based episomal vector carrying the tetracycline-regulated expression units, transactivator (rtTA2-M2), and tetO sequence. This Tet-on vector was kindly provided by Prof. Miwa (Tsukuba University, Japan).

### siRNAs

The on-target siRNAs were synthesized and purified from Dharmacon and include the following target sequences: siTERRA-1 (5′-NNAGGGUUAGGGUUAGGGUUA-3′), siTERRA-2 (5′-NNGGGUUAGGGUUAGGGUUAG-3′), and control (Dharmacon nontargeting siRNA D-001810-01). Cells were transfected with 100 nM siRNAs using Lipofectamine RNAiMAX (Invitrogen Corp.)

### Mammalian cell cultivation and preparation for live cell imaging

U2OS cells were cultured in Dulbecco’s modified Eagle’s medium (DMEM) (Gibco, MA) containing 10% fetal bovine serum (FBS) (Gibco, MA) at 37 °C in a 5% CO_2_ atmosphere. We used a retrovirus to generate U2OS cells that stably expressed iRFP-TRF1. The expression vector iRFP-TRF1 (iRFP-TRF1/pCLNCX) and an envelope plasmid were transfected into Gag293 cells with Lipofectamine 2000 (Invitrogen Corp., CA). The medium was refreshed 8 h after the transfection. The supernatant containing viral particles was mixed with 50% fresh medium and polybrene 24 h after the initial transfection. The mixture was placed on the target cells and was refreshed after 4 h.

For fluorescence imaging, the cells were plated on glass-bottomed dishes (Asahi Glass Co., Japan). The cells were transfected with the expression plasmids using Lipofectamine LTX (Invitrogen Corp.) according to the manufacturer’s protocol. The cells were then incubated with 0.1-μM TMR for 0.5 h at 37 °C to label the SNAPf tag. Unbound TMR was washed away with DMEM containing 10% FBS. Before the observation, the cell cultivation medium was replaced by Hanks’ balanced salt solution (HBSS) (Gibco, MA) containing minimum essential medium (MEM) amino acids solution (Gibco), non-essential amino acids solution (NEAA) (Gibco), 2.5-g/l glucose, 2-mM glutamine, 1-mM sodium pyruvate, and 10-mM 4-(2-hydroxyethyl)-1-piperazine ethanesulfonic acid (HEPES) (pH 7.4). For the heat shock experiment, the cells were incubated at 42 °C for 1 hour before the data acquisition. For the doxycycline treatment, the cells were treated with doxycycline 10 ng/mL for 2 hr.

### RNA isolation and reverse transcription quantitative real-time polymerase chain reaction (RT-qPCR)

Cells transfected with siControl or siTERRA-1 or -2 were treated with RNAiso Plus (TAKARA, Japan) and were collected to a centrifuge tube containing YTZ ball with a diameter of 5 mm (NIKKATO, Japan). Cells were homogenized by oscillating the tube at the speed of 50/sec for 2 min. After the homogenization of the cells, CHCl_3_ were added to the tubes. The tubes were centrifuged at 12,000 g for 15 min at 4 °C. The top layers were transferred to new tubes and added an equal amount of isopropanol. After the vortexing, the tubes were centrifuged at 12,000 g for 15 min at 4 °C and the supernatant were removed. 75% cold ethanol was added to the tubes. The tubes were centrifuged at 12,000 g for 1 min at 4 °C and supernatants were discarded. After the precipitate is dry, isolated RNA was dissolved by RNase-free water.

Isolated RNA was reverse transcribed into cDNA using the SuperScript 4 (Invitrogen Corp.). The cDNA was amplified using the primer set listed in Table S2^37^. SYBR Premix Ex Taq2 (Perfect Real Time) (TaKaRa) was used according to the manufacturer’s instructions. Quantitative real-time reverse transcription polymerase chain reaction (RT-qPCR) analysis was performed using a Thermal Cycler Dies Real Time system (TaKaRa).

### Fluorescence *in situ* hybridization

The U2OS cells on glass-bottomed dishes were incubated in ice-cold cytoskeleton (CSK) buffer (100-mM NaCl, 300-mM sucrose, 3-mM MgCl_2_, 10-mM piperazine-N,N’-bis(2-ethanesulfonic acid) [PIPES] pH 7, and 0.5% Triton X-100) containing 10-mM vanadyl ribonucleoside complex (NEB, NA) for 7 min. After being washed in phosphate-buffered saline (PBS), the cells were treated with 4% paraformaldehyde (PFA) in PBS (pH 7) for 10 min for chemical fixation and then rinsed in 70% ethanol at room temperature. The cells were dehydrated through treatment with an ethanol series (70%, 85%, and 100% ethanol for 5 min each) at room temperature and were then air dried. The TERRA molecules in the cells were hybridized with 5′-TMR-labeled antisense oligonucleotide (TMR-(GGGTTA)_7_) using 40 μl of a mixture containing 2 × saline sodium citrate buffer (SSC), 2-mg/ml bovine serum albumin (BSA), 10% dextran sulfate, 20% formamide and 10-mM vanadyl ribonucleoside complex for 16 h at 37 °C. Cell samples were washed three times in 2 × SSC containing 50% formamide (pH 7) for 5 min at 39 °C, three times in 2 × SSC (pH 7) for 5 min at 39 °C, and once in 2 × SSC (pH 7) for 5 min at room temperature.

### Metaphase telomere FISH

FISH analysis on metaphase spreads were performed as previously described^19^. Briefly, U2OS cells transfected with empty vector or the TERRA probe were treated with colcemid (0.1 μg/ml; Gibco) for 2 hr to accumulate mitotic cells. Then the cells are trypsinized, resuspended in 75 mM KCl hypotonic solution at 37 °C for 30 min, and fixed in fresh 3:1 methanol/acetic acid (v/v) for 4 times. Fixed cells were dropped onto wet glass microscope slides and allowed to dry for overnight. Metaphase spreads were stained with 0.1 μg/ml DAPI and analyzed by FISH using PNA-telomere probe according to manufacturer’s instruction (DakoCytomation, Denmark).

### Immunostainings

Chemical fixation of U2OS cells was performed by incubation in 2% PFA in PBS for 5 min at room temperature. The cells were then permeabilized by treatment in 0.1% sodium citrate/0.1% Triton X-100 for 5 min at room temperature. Blocking was performed in PBS containing 5% (w/v) BSA and 0.1% Tween-20 for 60 min at room temperature. The cells were stained with mouse anti-GFP IgG (Roche, Germany) as the primary antibody for 60 min at room temperature and then with AlexaFluor 568-conjugated goat anti-mouse IgG (Invitrogen Corp., CA) as the secondary antibody. The cells were then washed with PBS containing 0.5% (w/v) BSA and 0.1% Tween-20.

### Purification of mPUMt

The mPUMt gene was subcloned into a pTYB3 vector (NEB, NA) to produce an N-terminal fusion with an intein/chitin-binding domain. The gene for the fusion protein in the pTYB3 plasmid was expressed in the *Escherichia coli* BL21 strain. The bacterial body was sonicated in 20-mM sodium phosphate buffer (pH 8.0) containing 1-M NaCl and 0.1-mM phenylmethylsulfonyl fluoride (PMSF). The obtained lysate was clarified by centrifugation and incubated with chitin beads (NEB, NA) for 40 min. The beads were washed twice with 20-mM sodium phosphate (pH 8.0) buffer containing 1.0-M NaCl and 0.1-mM PMSF; once with 20-mM sodium phosphate (pH 8.0), 0.5-M NaCl, and 0.1-mM PMSF; and once with 20-mM sodium phosphate (pH 8.0), 0.15-M NaCl, and 0.1-mM PMSF. Cleavage of the fusion protein on the beads was initiated by adding 50-mM dithiothreitol (DTT). Before the incubation, oxygen was purged from the solution by exchanging with argon gas. The obtained recombinant mPUMt was collected and concentrated using Amicon Ultra-15 filter units (Merck Millipore, MA). The protein concentration in the yielded solution was determined using bicinchoninic acid (BCA) assay with BSA as a standard.

### RNA Electrophoretic Mobility Shift Assay

RNA oligonucleotides were purchased from Invitrogen Corp. and were radiolabeled at their 5′ ends using [γ-^32^P]ATP and T4 polynucleotide kinase (Toyobo Co. Ltd., Japan) following the manufacturer’s instructions. Various concentrations of the mPUMt protein and 5-nM radiolabeled RNA were incubated in binding buffer (10-mM HEPES, pH 7.4, 50-mM KCL, 1-mM EDTA, 0.01% Tween-20, 0.1-mg/ml BSA, and 1-mM DTT). For competition analysis, unlabeled oligonucleotides such as DNA with the sequence TTAGGGTT, RNA with the Nanos Response Element (NRE), or RNA with the sequence UUAGGGUU, were added to the binding mixture. The mixture was incubated for 1.5 h at room temperature and was subjected immediately to native polyacrylamide gel electrophoresis (PAGE) using a 6% polyacrylamide gel in 0.5% Tris/borate/ethylenediaminetetraacetic acid (EDTA) (TBE). The gels were dried and exposed to storage phosphor screens overnight and were then scanned (Fuji 9000; Fujifilm, Japan).

### Imaging data acquisition

Single-particle fluorescence imaging of TERRA was performed using a home-built total internal reflection fluorescence (TIRF) microscope system. This system was constructed on an inverted fluorescence microscope (IX81; Olympus Corp., Japan) equipped with laser lines at 488 nm (CYAN-488; Spectra-Physics, CA) and 561 nm (JUNO 561; SOC Corp., Japan) with a PlanApo 100× oil immersion objective with a numerical aperture of 1.49. The laser beams were placed off but parallel to the optical axis to obtain inclined excitation illumination^44^,^45^. Emissions from EGFP and TMR were collected by the objective and were captured by two scientific complementary metal-oxide semiconductor (sCMOS) cameras (ORCA-Flash4.0v2; Hamamatsu Photonics KK, Japan). Then iRFP fluorescence was detected using an electron-multiplying charge-coupled device (EM-CCD) camera (ImagEM; Hamamatsu Photonics KK). Images were acquired using software (MetaMorph; Molecular Devices Corp.) at a frame rate of 10 Hz.

### Image analysis

Positional differences between the cameras and image distortion resulting from optical aberrations in the fluorescence images were corrected using a pinhole array, as described previously by Koyama-Honda *et al*.^46^. The pinhole array contained a lattice of 1-μm-diameter holes at 5-μm intervals. A bright-field transmission image of the array was recorded on each camera. The detected pixel positions of the holes were used to determine a set of third-order spline functions, which describe the pixel-to-coordinate relation for each camera. This set of functions was then applied for undistortion of the raw fluorescence images to obtain corrected images.

Before analysis, image noise reduction was performed through Gaussian blur filtering with ImageJ software. The *x* and *y* coordinates of each fluorescent spot were determined using a homemade program based on the cross-correlation method, as described previously^30^,^47^,^48^,^49^. In this method, an image and a kernel are described, respectively, as intensity matrices **I** and **K**, which contain both fluorescence signals and background. We set **K** to exhibit a 2D Gaussian distribution with a FWHM of 200 nm for EGFP and TMR and 500 nm for iRFP. The image was scanned by the kernel in one-pixel increments. For each increment, the program calculated a correlation value that describes how well the values in the kernel match those of the underlying image. At the relative shift, the position at which the kernel and the image were most similar was a maximum in the correlation matrix (**X**). Here, the cross-correlation between the kernel (**K**) and the image (**I**) is given as shown below.


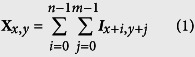


In that equation, *x* and *y* denote the distance that the kernel (**K**) has moved over the original image (**I**). In the correlation matrix (**X**), regions with correlation values exceeding those of the background and with areas larger than five pixels were regarded as fluorescent spots. Their positions were determined to be the centroids of the correlation values of the detected spots.

The localization precision of the detected EGFP and TMR spots was estimated from the standard deviations of individual spot positions in chemically fixed cells. To create images of TERRA, hnRNPA1, and telomere without background, Gaussian spots with FWHM of 200 nm were placed on a blank image at the coordinates at which the EGFP and TMR spots were detected. Also, iRFP spots are shown on the same image as Gaussian spots with FWHM of 500 nm.

TERRA foci were defined as the nucleus position in which more than 10 TERRA spots appeared during 8 seconds observation (green to yellow spots in Fig. 2b).

The radial distribution function, ρ(*r*), was defined as the time-averaged density of TERRA spots in a region within a distance of *r* − 0.25 to *r* (μm) from the center of a telomere. The ρ(*r*) function was calculated using the following equation (2).


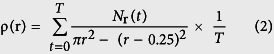


Therein, *N*_r_(*t*) is the number of detected TERRA spots in the region of *r* − *r* − 0.25 μm from a telomere at time *t. T* is the observation duration (8 s).

For each fluorescent spot’s trajectory, the mean square displacement (MSD) was calculated using the following equation (3).


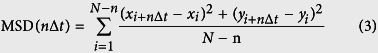


In that equation, ∆*t* is the sampling time interval, *x*_i_ and *y*_i_ are the *x* and *y* coordinates of the fluorescent spot at time *i*∆*t*, and *N* is the total frame number of the trajectory^50^,^51^. The diffusion coefficient was derived from the slope of the MSD-∆*t* plot for 0–300 ms using least-squares fitting. Because of the limitation of the localization precision, the positions of the EGFP spots in chemically fixed cells fluctuated from frame to frame, yielding an “apparent” diffusion coefficient. A histogram of the apparent diffusion coefficients was fitted with a log normal distribution function, from which the 99% confidence limit of the non-mobile spots was inferred. For classification of the fluorescent spot movements into stationary or mobile modes, spots with a diffusion coefficient smaller than the 99% confidence limit were judged as stationary. The remainders were classified as mobile.

An hnRNPA1 spot and a TERRA spot were regarded as colocalized if the distance between these spots was less than or equal to a threshold value. The threshold was determined as the sum of the localization precisions of EGFP and TMR and the overlay accuracy between the EGFP and TMR images. The localization precisions of EGFP and TMR were estimated from the standard deviations of the spot positions in chemically fixed cells as (σ_*x*_ + σ_*y*_)/2, where σ_*x*_ and σ_*y*_ are the standard deviations of the centroid positions in the *x* and *y* dimensions, respectively^52^. To evaluate the overlay accuracy of the images measured on different color channels, TetraSpeck Fluorescent Microspheres on the glass surface were compared after spatial correction. The differences in the centroid positions (∆*x* and ∆*y*) of the microspheres between color channels were calculated. The overlay accuracies were ascertained as (σ′_*x*_ + σ′_*y*_)/2, where σ′_*x*_ and σ′_*y*_ are the standard deviations of ∆x and ∆y, respectively. All calculations were done using IGOR Pro software (WaveMetrics Inc., Lake Oswego, OR).

## Additional Information

**How to cite this article**: Yamada, T. *et al*. Spatiotemporal analysis with a genetically encoded fluorescent RNA probe reveals TERRA function around telomeres. *Sci. Rep.* **6**, 38910; doi: 10.1038/srep38910 (2016).

**Publisher's note:** Springer Nature remains neutral with regard to jurisdictional claims in published maps and institutional affiliations.

## Supplementary Material

Supplementary Information

Supplementary video S1

Supplementary video S2

Supplementary video S3

Supplementary video S4

Supplementary video S5

## References

[b1] Moyzis R. K. . A highly conserved repetitive DNA sequence, (TTAGGG)n, present at the telomeres of human chromosomes. Proc. Natl. Acad. Sci. USA 85, 6622–6626 (1988). 3413114 10.1073/pnas.85.18.6622 PMC282029

[b2] Griffith J. D. . Mammalian Telomeres End in a Large Duplex Loop. Cell 97, 503–514 (1999). 10338214 10.1016/s0092-8674(00)80760-6

[b3] Blackburn E. H., Epel E. S. & Lin J. Human telomere biology: A contributory and interactive factor in aging, disease risks, and protection. Science 350, 1193–1198 (2015). 26785477 10.1126/science.aab3389

[b4] Levy M. Z., Allsopp R. C., Futcher A. B., Greider C. W. & Harley C. B. Telomere end-replication problem and cell aging. J. Mol. Biol. 225, 951–960 (1992). 1613801 10.1016/0022-2836(92)90096-3

[b5] Verdun R. E. & Karlseder J. Replication and protection of telomeres. Nature 447, 924–931 (2007). 17581575 10.1038/nature05976

[b6] Sarek G., Marzec P., Margalef P. & Boulton S. J. Molecular basis of telomere dysfunction in human genetic diseases. Nat. Struct. Mol. Biol. 22, 867–874 (2015). 26581521 10.1038/nsmb.3093

[b7] Nugent C. I. . Telomere maintenance is dependent on activities required for end repair of double-strand breaks. Curr. Biol. 8, 657–662 (1998). 9635193 10.1016/s0960-9822(98)70253-2

[b8] Orthwein A. . Mitosis Inhibits DNA Double-Strand Break Repair to Guard Against Telomere Fusions. Science 344, 189–193 (2014). 24652939 10.1126/science.1248024

[b9] Paeschke K., Simonsson T., Postberg J., Rhodes D. & Lipps H. J. Telomere end-binding proteins control the formation of G-quadruplex DNA structures *in vivo*. Nat. Struct. Mol. Biol. 12, 847–854 (2005). 16142245 10.1038/nsmb982

[b10] de Lange T. Shelterin: the protein complex that shapes and safeguards human telomeres. Genes Dev. 19, 2100–2110 (2005). 16166375 10.1101/gad.1346005

[b11] Masutomi K. . Telomerase Maintains Telomere Structure in Normal Human Cells. Cell 114, 241–253 (2003). 12887925 10.1016/s0092-8674(03)00550-6

[b12] Azzalin C. M., Reichenbach P., Khoriauli L., Giulotto E. & Lingner J. Telomeric Repeat–Containing RNA and RNA Surveillance Factors at Mammalian Chromosome Ends. Science 318, 798–801 (2007). 17916692 10.1126/science.1147182

[b13] Schoeftner S. & Blasco M. A. Developmentally regulated transcription of mammalian telomeres by DNA-dependent RNA polymerase II. Nat. Cell Biol. 10, 228–236 (2007). 18157120 10.1038/ncb1685

[b14] Sandell L. L., Gottschling D. E. & Zakian V. A. Transcription of a yeast telomere alleviates telomere position effect without affecting chromosome stability. Proc. Natl. Acad. Sci. USA 91, 12061–12065 (1994). 7991584 10.1073/pnas.91.25.12061 PMC45376

[b15] Solovei I., Gaginskaya E. R. & Macgregor H. C. The arrangement and transcription of telomere DNA sequences at the ends of lampbrush chromosomes of birds. Chromosome Res. 2, 460–470 (1994). 7834223 10.1007/BF01552869

[b16] Rippe K. & Luke B. TERRA and the state of the telomere. Nat. Struct. Mol. Biol. 22, 853–858 (2015). 26581519 10.1038/nsmb.3078

[b17] Scheibe M. . Quantitative interaction screen of telomeric repeat-containing RNA reveals novel TERRA regulators. Genome Res. 23, 2149–2157 (2013). 23921659 10.1101/gr.151878.112 PMC3847783

[b18] de Silanes I. L., d’Alcontres M. S. & Blasco M. A. TERRA transcripts are bound by a complex array of RNA-binding proteins. Nat. Commun. 1, 33 (2010). 20975687 10.1038/ncomms1032

[b19] Deng Z., Norseen J., Wiedmer A., Riethman H. & Lieberman P. M. TERRA RNA Binding to TRF2 Facilitates Heterochromatin Formation and ORC Recruitment at Telomeres. Mol. Cell 35, 403–413 (2009). 19716786 10.1016/j.molcel.2009.06.025 PMC2749977

[b20] Flynn R. L. . TERRA and hnRNPA1 orchestrate an RPA-to-POT1 switch on telomeric single-stranded DNA. Nature 471, 532–536 (2011). 21399625 10.1038/nature09772 PMC3078637

[b21] Arnoult N., Van Beneden A. & Decottignies A. Telomere length regulates TERRA levels through increased trimethylation of telomeric H3K9 and HP1α. Nat. Struct. Mol. Biol. 19, 948–956 (2012). 22922742 10.1038/nsmb.2364

[b22] Ozawa T., Natori Y., Sato M. & Umezawa Y. Imaging dynamics of endogenous mitochondrial RNA in single living cells. Nat. Meth. 4, 413–419 (2007). 10.1038/nmeth1030 17401370

[b23] Yamada T., Yoshimura H., Inaguma A. & Ozawa T. Visualization of Nonengineered Single mRNAs in Living Cells Using Genetically Encoded Fluorescent Probes. Anal. Chem. 83, 5708–5714 (2011). 21634804 10.1021/ac2009405

[b24] Yoshimura H., Inaguma A., Yamada T. & Ozawa T. Fluorescent Probes for Imaging Endogenous β-Actin mRNA in Living Cells Using Fluorescent Protein-Tagged Pumilio. ACS Chem. Biol. 7, 999–1005 (2012). 22387832 10.1021/cb200474a

[b25] Wang X., McLachlan J., Zamore P. D. & Hall T. M. T. Modular Recognition of RNA by a Human Pumilio-Homology Domain. Cell 110, 501–512 (2002). 12202039 10.1016/s0092-8674(02)00873-5

[b26] Cheong C.-G. & Hall T. M. T. Engineering RNA sequence specificity of Pumilio repeats. Proc. Natl. Acad. Sci. USA 103, 13635–13639 (2006). 16954190 10.1073/pnas.0606294103 PMC1564246

[b27] Filipovska A., Razif M. F. M., Nygård K. K. A. & Rackham O. A universal code for RNA recognition by PUF proteins. Nat. Chem. Biol. 7, 425–427 (2011). 21572425 10.1038/nchembio.577

[b28] Wang X. . Rapid telomere motions in live human cells analyzed by highly time-resolved microscopy. Epigenetics Chromatin 1, 4 (2008). 19014413 10.1186/1756-8935-1-4 PMC2585561

[b29] Arora R., Brun C. M. & Azzalin C. M. Transcription regulates telomere dynamics in human cancer cells. RNA 18, 684–693 (2012). 22357912 10.1261/rna.029587.111 PMC3312556

[b30] Fujiwara T., Ritchie K., Murakoshi H., Jacobson K. & Kusumi A. Phospholipids undergo hop diffusion in compartmentalized cell membrane. J. Cell Biol. 157, 1071–1082 (2002). 12058021 10.1083/jcb.200202050 PMC2174039

[b31] Le P. N., Maranon D. G., Altina N. H., Battaglia C. L. R. & Bailey S. M. TERRA, hnRNP A1, and DNA-PKcs Interactions at Human Telomeres. Front. Oncol. 3, 91 (2013). 23616949 10.3389/fonc.2013.00091 PMC3628365

[b32] Saxton M. J. Single-particle tracking: the distribution of diffusion coefficients. Biophys. J. 72, 1744–1753 (1997). 9083678 10.1016/S0006-3495(97)78820-9 PMC1184368

[b33] Lateral diffusion of membrane-spanning and glycosylphosphatidylinositol- linked proteins: toward establishing rules governing the lateral mobility of membrane proteins. *J. Cell Biol.* **115**, 75–84 (1991). 10.1083/jcb.115.1.75 PMC2289918 1680869

[b34] Politz J. C., Tuft R. A., Pederson T. & Singer R. H. Movement of nuclear poly(A) RNA throughout the interchromatin space in living cells. Curr. Biol. 9, 285–291 (1999). 10209094 10.1016/s0960-9822(99)80136-5

[b35] Ishihama Y. & Funatsu T. Single molecule tracking of quantum dot-labeled mRNAs in a cell nucleus. Biochem. Biophys. Res. Commun. 381, 33–38 (2009). 19351590 10.1016/j.bbrc.2009.02.001

[b36] Hewitt G. . Telomeres are favoured targets of a persistent DNA damage response in ageing and stress-induced senescence. Nat. Commun. 3, 708, (2012). 22426229 10.1038/ncomms1708 PMC3292717

[b37] Feretzaki M. & Lingner J. A practical qPCR approach to detect TERRA, the elusive telomeric repeat-containing RNA. Methods in press. 10.1016/j.ymeth.2016.08.004 27530378

[b38] Collie G. W., Haider S. M., Neidle S. & Parkinson G. N. A crystallographic and modelling study of a human telomeric RNA (TERRA) quadruplex. Nucleic Acids Res. 38, 5569–5580 (2010). 20413582 10.1093/nar/gkq259 PMC2938214

[b39] Biffi G., Di Antonio M., Tannahill D. & Balasubramanian S. Visualization and selective chemical targeting of RNA G-quadruplex structures in the cytoplasm of human cells. Nat. Chem. 6, 75–80 (2014). 24345950 10.1038/nchem.1805 PMC4081541

[b40] Azzalin C. M. & Lingner J. Telomere functions grounding on TERRA firma. Trends Cell Biol. 25, 29–36 (2015). 25257515 10.1016/j.tcb.2014.08.007

[b41] Porro A. . Functional characterization of the TERRA transcriptome at damaged telomeres. Nat. Commun. 5 (2014). 10.1038/ncomms6379 PMC4264578 25359189

[b42] Redon S., Zemp I. & Lingner J. A three-state model for the regulation of telomerase by TERRA and hnRNPA1. Nucleic Acids Res. 41, 9117–9128 (2013). 23935072 10.1093/nar/gkt695 PMC3799450

[b43] Akagi T., Sasai K. & Hanafusa H. Refractory nature of normal human diploid fibroblasts with respect to oncogene-mediated transformation. Proc. Natl. Acad. Sci. USA 100, 13567–13572 (2003). 14597713 10.1073/pnas.1834876100 PMC263854

[b44] Tokunaga M., Imamoto N. & Sakata-Sogawa K. Highly inclined thin illumination enables clear single-molecule imaging in cells. Nat. Meth. 5, 159–161 (2008). 10.1038/nmeth1171 18176568

[b45] Izeddin I. . Single-molecule tracking in live cells reveals distinct target-search strategies of transcription factors in the nucleus. eLife 3 (2014). 10.7554/eLife.02230 PMC4095940 24925319

[b46] Koyama-Honda I. . Fluorescence Imaging for Monitoring the Colocalization of Two Single Molecules in Living Cells Biophys. J. 81, 2378–2388 (2001). 15596511 10.1529/biophysj.104.048967 PMC1305264

[b47] Gelles J., Schnapp B. J. & Sheetz M. P. Tracking kinesin-driven movements with nanometre-scale precision. Nature 331, 450–453 (1988). 3123999 10.1038/331450a0

[b48] Suzuki K. G. N. . GPI-anchored receptor clusters transiently recruit Lyn and Gα for temporary cluster immobilization and Lyn activation: single-molecule tracking study 1. J. Cell Biol. 177, 717–730 (2007). 17517964 10.1083/jcb.200609174 PMC2064216

[b49] Cheezum M. K., Walker W. F. & Guilford W. H. Quantitative comparison of algorithms for tracking single fluorescent particles. Biophys. J. 81, 2378–2388 (2001). 11566807 10.1016/S0006-3495(01)75884-5 PMC1301708

[b50] Qian H., Sheetz M. P. & Elson E. L. Single particle tracking. Analysis of diffusion and flow in two-dimensional systems. Biophys. J. 60, 910–921 (1991). 1742458 10.1016/S0006-3495(91)82125-7 PMC1260142

[b51] Saxton M. J. & Jacobson K. Single-particle tracking: applications to membrane dynamics. Annu.Rev. Biophys. Biomol. Struct. 26, 373–399 (1997). 9241424 10.1146/annurev.biophys.26.1.373

[b52] Rust M. J., Bates M. & Zhuang X. Sub-diffraction-limit imaging by stochastic optical reconstruction microscopy (STORM). Nat Meth 3, 793–796 (2006). 10.1038/nmeth929 PMC2700296 16896339

