# Supplementary material for: Spatiotemporal analysis with a genetically encoded fluorescent RNA probe reveals TERRA function around telomeres
Source: Sci Rep. 2016 Dec 13;6:38910. doi: 10.1038/srep38910 (PMC5153658; doi:10.1038/srep38910)
Supplement: Supplementary Information [file srep38910-s1.pdf]

# Supplementary Information

## Spatiotemporal analysis with a genetically encoded fluorescent RNA probe reveals TERRA function around telomeres

Toshimichi Yamada[1], Hideaki Yoshimura[1], Rintaro Shimada[1], Mitsuru Hattori[1], Masatoshi Eguchi[1], Takahiro K. Fujiwara[2], Akihiro Kusumi[2], Takeaki Ozawa[1,*]

[1]Department of School of Science, The University of Tokyo, 7-3-1 Hongo, Bunkyo-ku, Tokyo 113-0033, Japan

[2]Institute for Integrated Cell-Material Science (WPI-iCeMS), Kyoto University, Kyoto 606-8507, Japan

[*]**Correspondence to:** ozawa@chem.s.u-tokyo.ac.jp (T.O.)

**Supplementary results contain:**

Supplementary Figures 1-9

Supplementary Table 1-2

Video 1-5

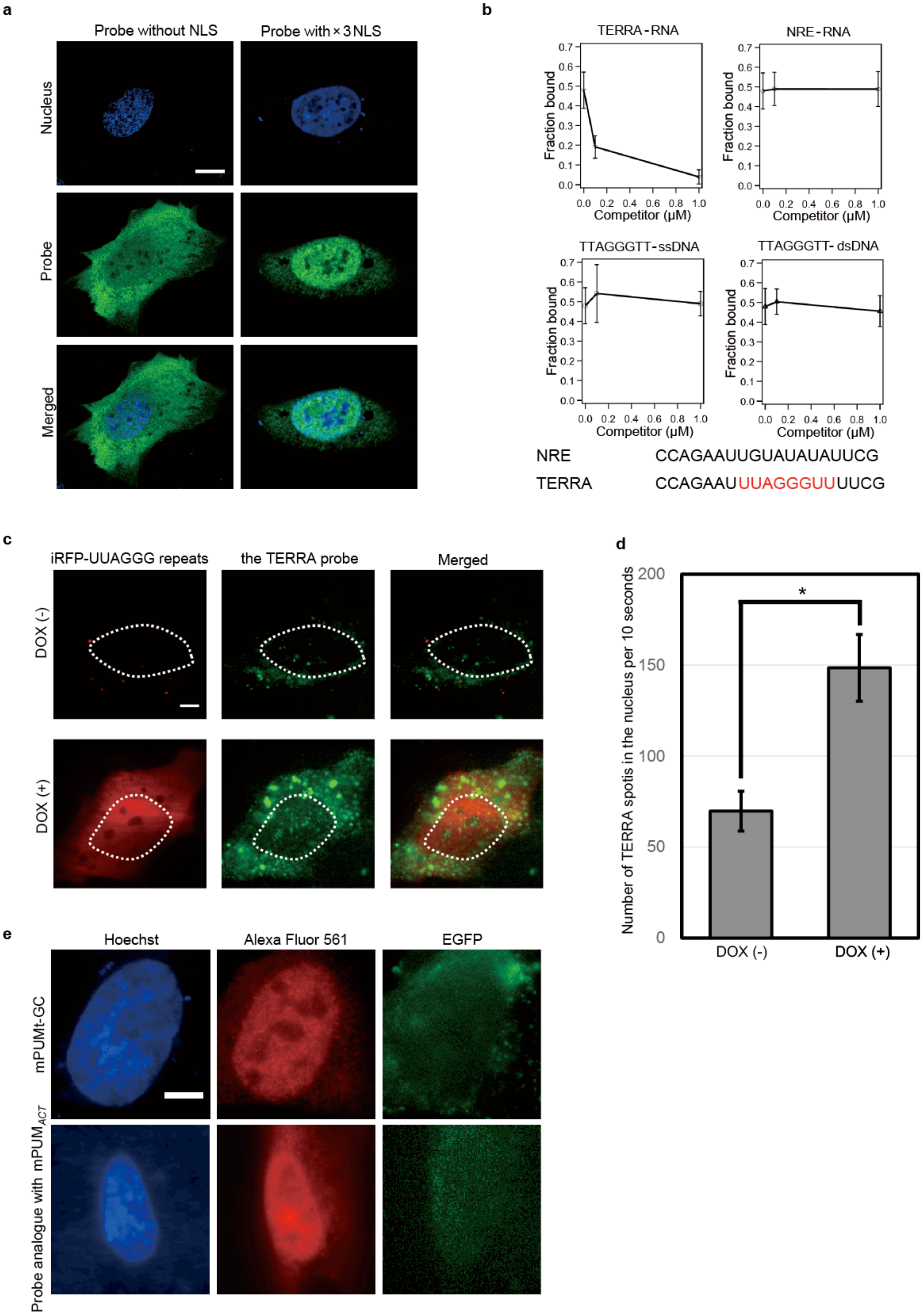
a
Probe without NLS
Probe with × 3NLS
Nucleus
Probe
Merged
b
TERRA-RNA
NRE-RNA
TTAGGGTT-ssDNA
TTAGGGTT- dsDNA
Fraction bound
Competitor (μM)
NRE CCAGAAUUGUAUAUAUUCG
TERRA CCAGAAUUUAGGGUUUUCG
c
iRFP-UUAGGG repeats
the TERRA probe
Merged
DOX (-)
DOX (+)
d
Number of TERRA spotis in the nucleus per 10 seconds
DOX (-)
DOX (+)
*
e
Hoechst
Alexa Fluor 561
EGFP
mPUMt-GC
Probe analogue with mPUM$_{ACT}$

**Supplementary Figure 1.** Characterization of the TERRA probe

(**a**) Localization of probes in the cells. Fluorescence images were obtained using fixed U2OS cells expressing the TERRA probe. Alexa Fluor 488-conjugated anti-GFP antibody was used for immunostaining to label the probe with the fluorescent molecule in the cells. DNA was stained with Hoechst 33342. The probe analog without NLS was distributed in the cytoplasm (left), whereas the probe with NLS repeats localized to the nucleus (right). Scale bar, 8.0 μm. (**b**) Analysis of selective mPUMt binding to target RNA. Competition assays of mPUMt binding to RI-labeled 5'-UUAGGGUU-3' RNA. The competition assays were performed in the presence of increasing amounts (0-, 1-, and 10-fold molar excess) of unlabeled oligonucleotide. Four competitors were used: UUAGGGUU-RNA, Nanos Response Element (NRE) RNA, and single-stranded and double-stranded TTAGGGT-DNA. The average bound fraction for each condition was shown against the concentration of each competitor, as indicated. (**c, d**) EGFP reconstitution of the TERRA probe with UUAGGG repeats. (**c**) Fluorescence images were obtained from U2OS cells expressing iRFP-$(UUAGGG)_{13}$ (left), the TERRA probe (middle), and the merged image (right). Nucleus is indicated with white broken line. Scale bar, 5.0 μm. (**d**) Quantification of the number of TERRA spots in the nucleus in the cells without dox treatment (DOX(-)) and with dox treatment (DOX(+)).*P*-value was computed using the Student's *t*-test. * indicates $P < 0.05$. (**e**) Analysis of spontaneous EGFP-fragment reconstitution with probe analogs. Fluorescence images were obtained using fixed U2OS cells expressing probe analogs. The cells were immunostained with an anti-GFP antibody, which binds to full-length EGFP and the C-terminal fragment of EGFP. Alexa Fluor 561-labeled secondary antibody was used to visualize the probe localization. DNA was stained with Hoechst 33342. When the analog lacking the N-terminal fragment of EGFP and the NLS partially localized to the nucleus (upper), no fluorescence signal for EGFP was detected. Similarly, upon expression of an analog containing $mPUM_{ACT}$, no fluorescence signal for EGFP was observed (bottom). Scale bar, 2.0 μm.

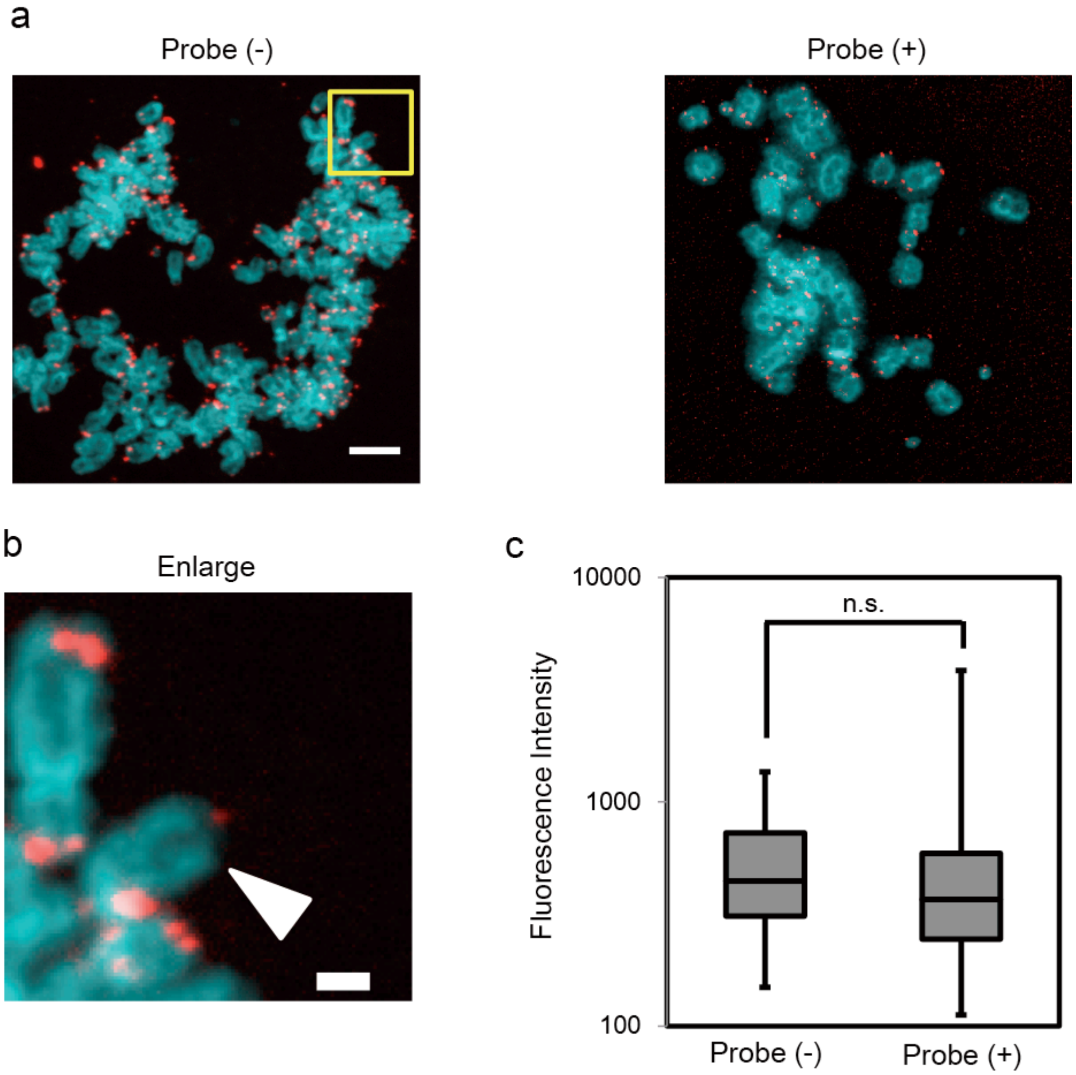


**Supplementary Figure 2**. The effect of the TERRA probe on telomere length and structure.

(**a**) Representative telomere quantitative FISH on metaphase spreads for U2OS cells in the absence (left panel) or presence (right panel) of the TERRA probe. Metaphase spreads were stained for telomeric DNA (red) and counterstained with DAPI (cyan). Scale bar, 10.0 µm. (**b**) An enlarged image of chromosomes with telomere free end (white arrowhead). Scale bar, 1.0 µm. (**c**) Quantification of fluorescence intensity of the telomere for U2OS cells in the presence or absence of the TERRA probe. *P*-value was computed using the Student's *t*-test. n.s. indicates $P > 0.05$.

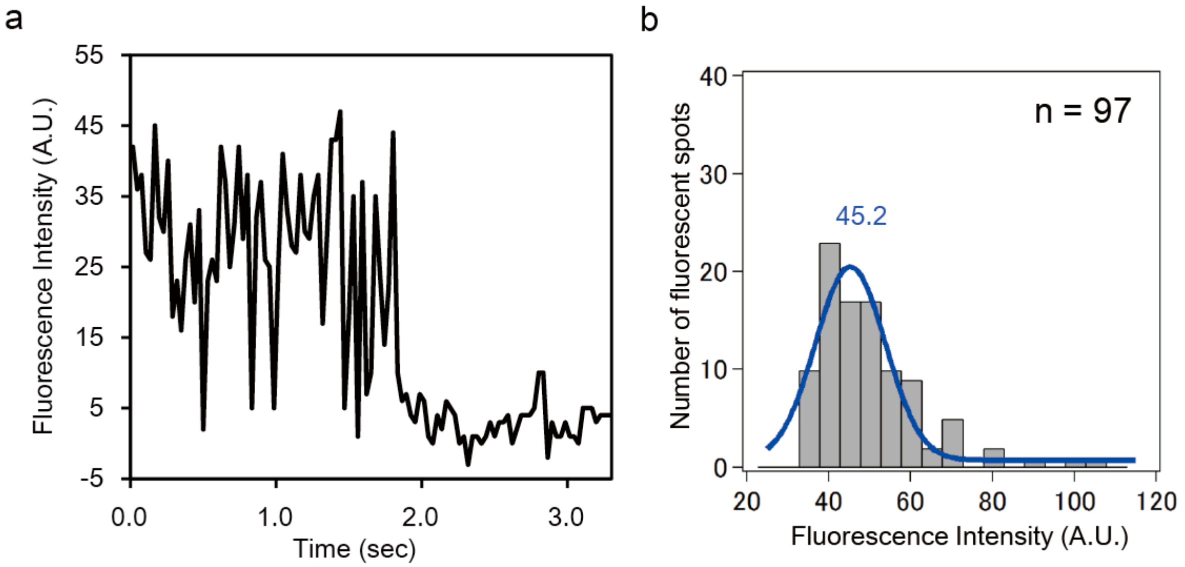


**Supplementary Figure 3.** Imaging a single TERRA molecule in live cells

(**a**) Time dependency of fluorescence intensity on a single EGFP fluorescent spot. A single-step photobleaching occurred at 1.8 sec. (**b**) Histogram showing fluorescence intensity distribution of individual spots in live cells. The histograms were fitted to a Gaussian distribution (blue line) with a peak value 45.2.

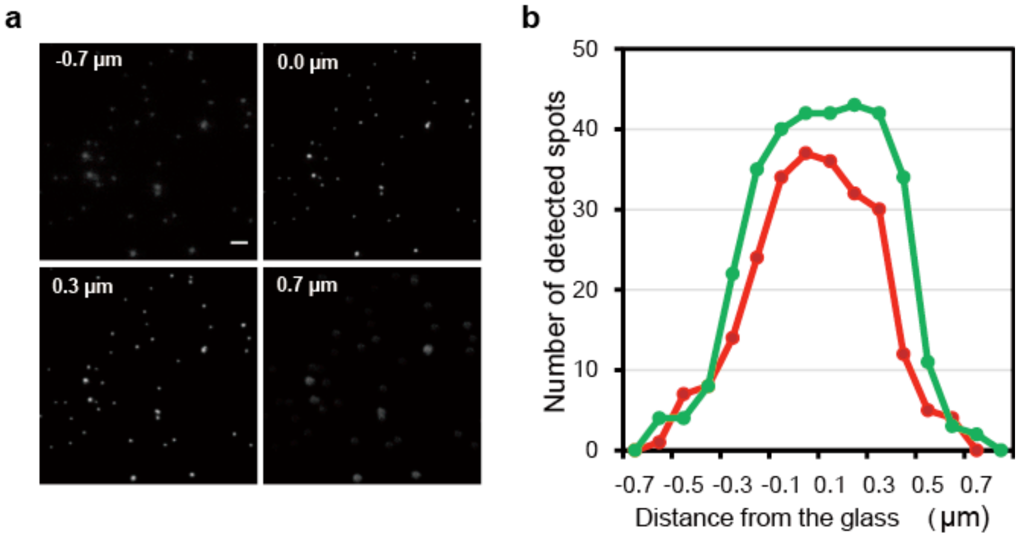


**Supplementary Figure 4.** Determination of the detection ranges in the z-axis direction using fluorescent beads.

(**a**) Images of multicolor fluorescent beads at different focus positions. In each image, the focus position is shown at the upper left (the glass surface is set as 0.0 µm). Scale bar, 2.0 µm. (**b**) Numbers of detected beads at various focus positions. More than 90% of the beads were detected within ± 200 nm of the surface, but the detection efficiency markedly decreased beyond this region.

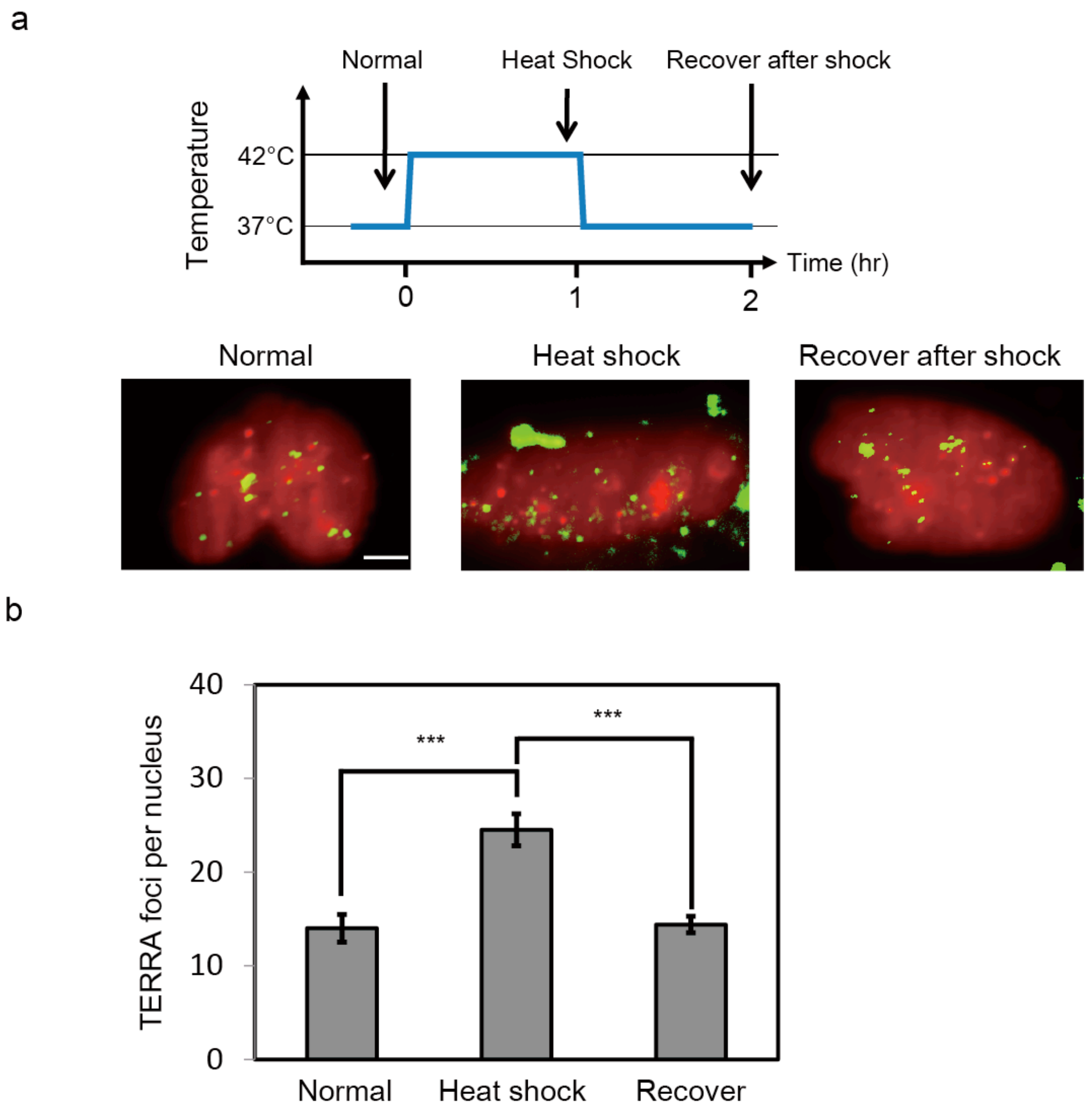


**Supplementary Figure 5.** Heat shock response of TERRA

(**a**) Scheme of the heat shock experiment. The distribution of TERRA foci constructed from the time integrated image of the movie. TERRA foci are shown in green; telomere in red. The scale bar represents 4.0 μm. (**b**) Number of TERRA foci in different temperature. Mean numbers of TERRA foci per nucleus (mean ± s.e.m) of each condition are indicated. *P*-value was computed using the Student's *t*-test. *** indicates $P < 0.001$.

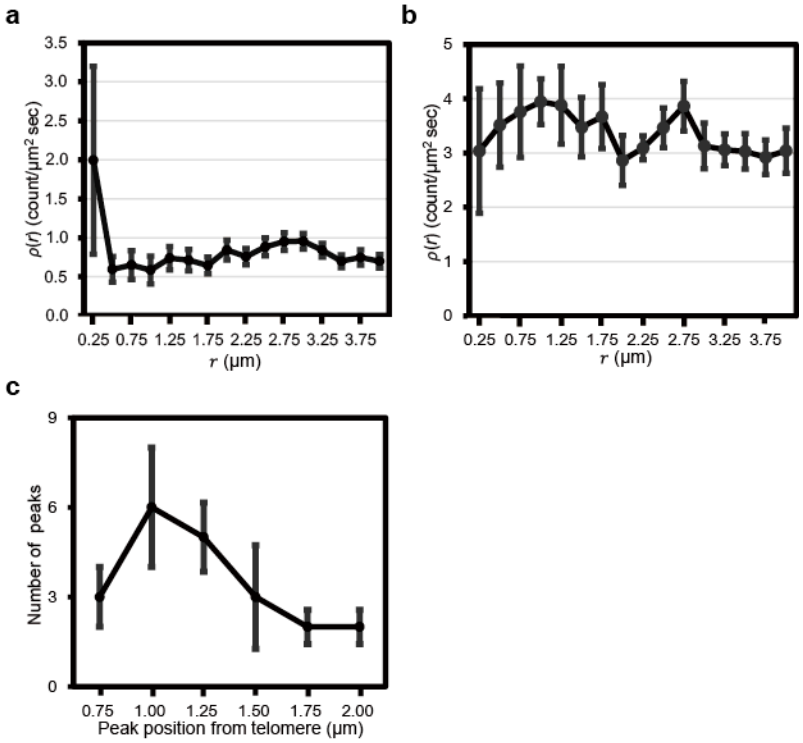


**Supplementary Figure 6.** Averaged ρ($r$) and the distribution of the number of ρ($r$) peaks.

(**a**) Averaged ρ($r$) of the TERRA spots in Figure 2b (±s.e.m., $n$ = 30) for randomly selected positions. When a position was selected at a TERRA-accumulated spot, ρ($r$ = 0.25 μm) exceeded ρ($r$ > 0.25 μm). (**b**) Averaged ρ($r$) of hnRNPA1 (±s.e.m., $n$ = 26) for the telomeres in Figure 2b. (**c**) Values for peak positions in ρ($r$) ($n$ = 117) in single cells are shown as a function of the distance from a telomere.

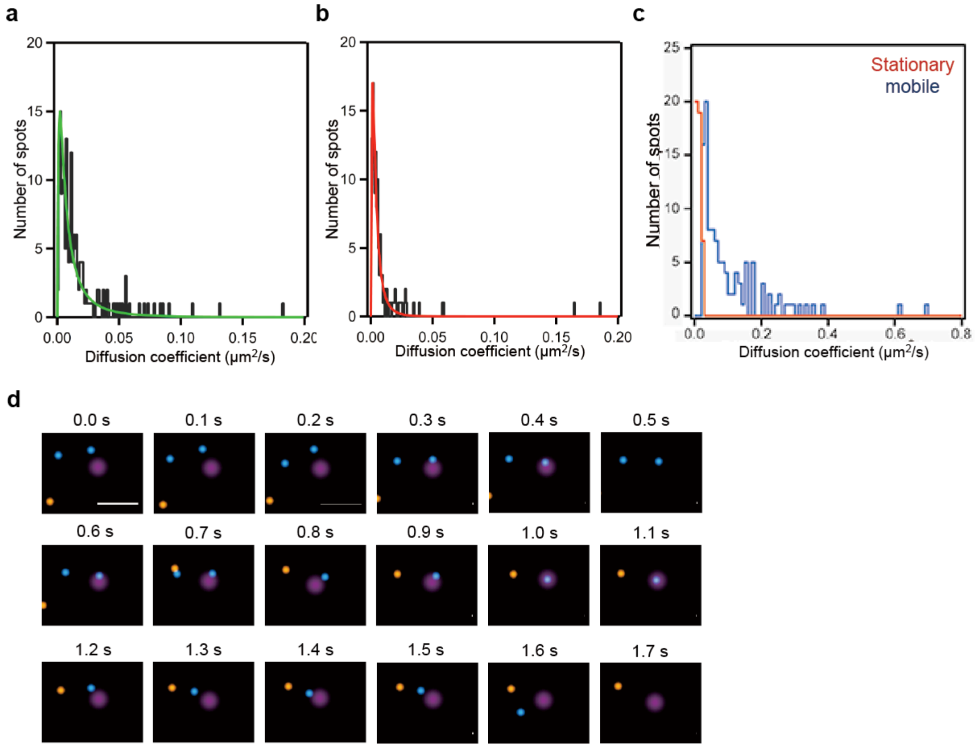


**Supplementary Figure 7.** Analysis of diffusion coefficients of fixed spots and hnRNPA1.

(**a**, **b**) Histogram of the diffusion coefficients of fluorescent spots in chemically fixed cells. The distribution of the apparent diffusion coefficients of EGFP spots (a, $n$ = 181) and TMR spots (b, $n$ = 141) in chemically fixed cells. Histograms are fitted with lognormal distributions with mean values of $5.32\times10^{-3}$ μm$^2$/s (variance, $9.33\times10^{-3}$ μm$^2$/s) for EGFP and $2.27\times10^{-3}$ μm$^2$/s (variance, $3.64\times10^{-3}$ μm$^2$/s) for TMR. (**c**, **d**) Analysis of hnRNPA1 dynamics at telomeres. (**c**) Distributions of the diffusion coefficients of stationary hnRNPA1 (orange, $n$ = 46) and mobile hnRNPA1 (cyan, $n$ = 112) on telomeres. (**d**) Sequential images of mobile hnRNPA1 localized transiently to a telomere (magenta). Scale bar, 1.0 μm.

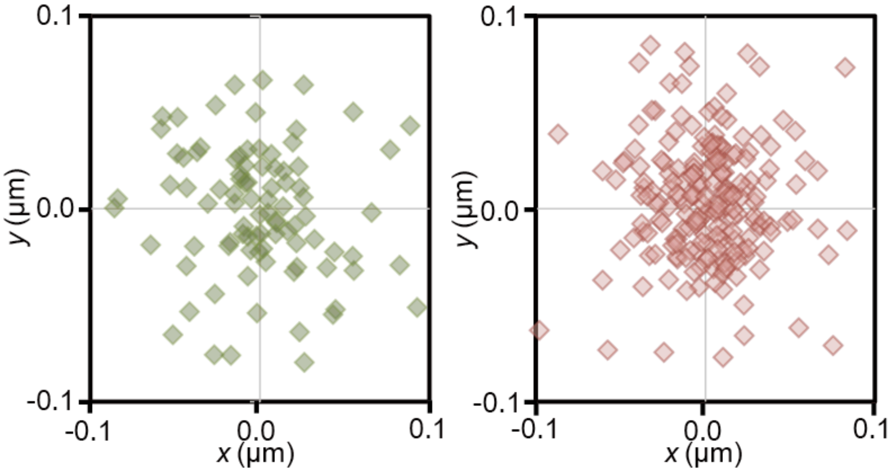


**Supplementary Figure 8.** Localization accuracy of fluorescent dyes.

Distribution of the centroid positions of individual EGFP (left) and TMR (right) spots in chemically fixed cells. Each spot gives a cluster of localizations because of the detection error during observations. The localizations of 95 clusters of EGFP and 205 clusters of TMR (indicated as diamonds) were aligned by their centers of mass to generate the overall 2D presentation of the localization distribution.

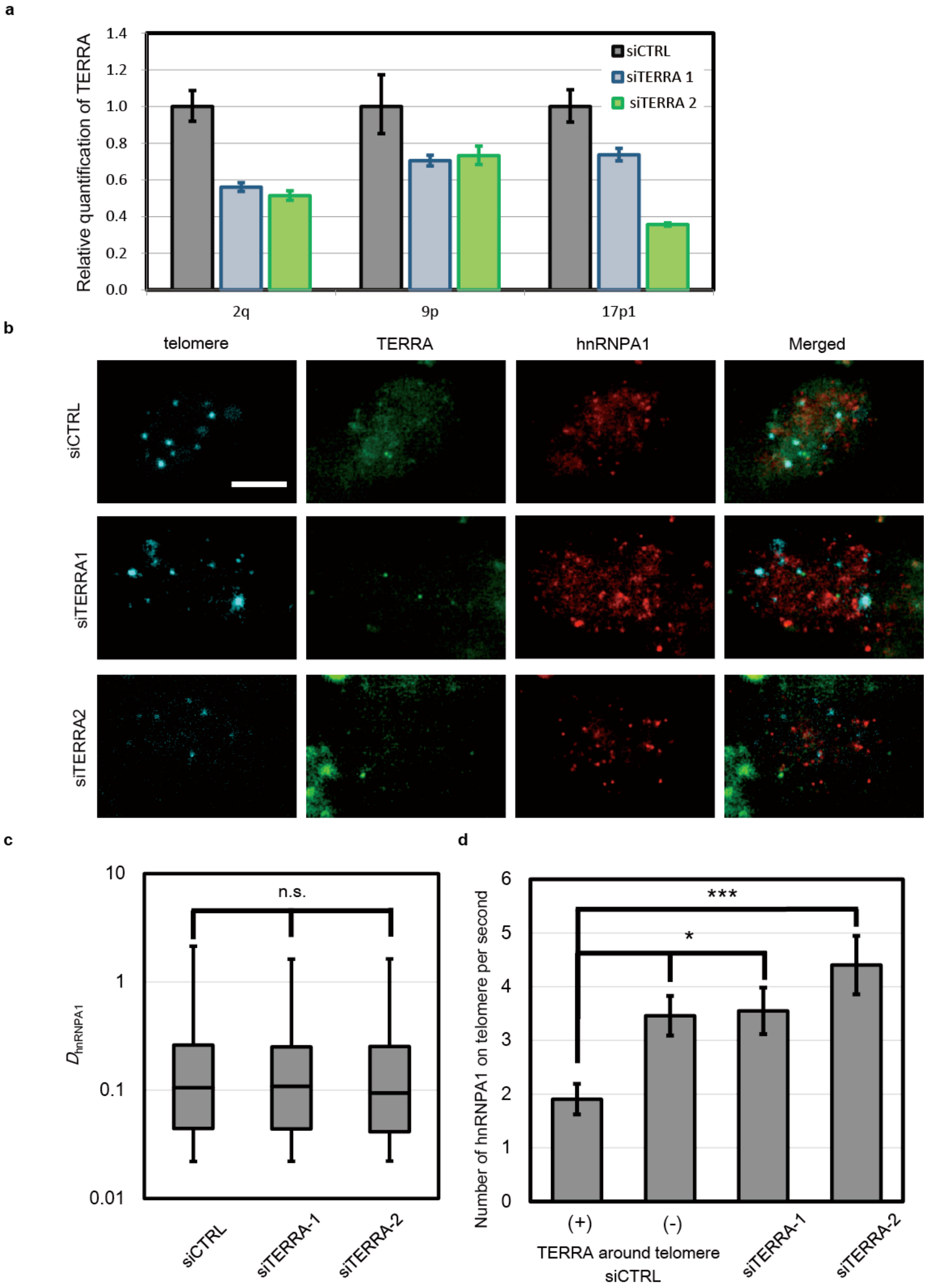
a
Relative quantification of TERRA
1.4
1.2
1.0
0.8
0.6
0.4
0.2
0.0
siCTRL
siTERRA 1
siTERRA 2
2q
9p
17p1
b
telomere
TERRA
hnRNPA1
Merged
siCTRL
siTERRA1
siTERRA2
c
10
1
0.1
0.01
n.s.
$D_{hnRNPA1}$
siCTRL
siTERRA-1
siTERRA-2
d
Number of hnRNPA1 on telomere per second
6
5
4
3
2
1
0
***
*
(+)
(-)
TERRA around telomere
siCTRL
siTERRA-1
siTERRA-2

**Supplementary Figure 9.** The effect of TERRA depletion on hnRNPA1 motion and distribution.

(**a**) U2OS cells were transfected with siCTRL, siTERRA1, and siTERRA2. The amount of TERRA from individual subtelomeres were measured from total RNA and normalized to GAPDH mRNA. The bars represent the average values from three biological and two technical replicates for each sample. (**b**) Fluorescence images were obtained from U2OS cells expressing iRFP-TRF1 (telomere), the TERRA probe (TERRA), SNAP-hnRNPA1 labeled with TMR (hnRNPA1), and their merged images. The cells were transfected with siCTRL (upper), siTERRA-1 (middle), and siTERRA-2 (right). The scale bar represents 5.0 μm. (**c**) Quantification of the diffusion coefficients of hnRNPA1 in the cells transfected with each siRNA. n = 513 (siCTRL), 592 (siTERRA-1), 376 (siTERRA-2). (**d**) The number of hnRNPA1 spots observed at the telomeres in each cell condition. Mean numbers of hnRNPA1 spots per second (mean ± s.e.m) under each condition are indicated. In **c** and **d**, $P$-value was computed using the Student's $t$-test. n.s. indicates $P > 0.05$, * indicates $P < 0.05$, *** indicates $P < 0.001$.

| Repeats | R8 | R7 | R6 | R5 | R4 | R3 | R2 | R1 |
|---|---|---|---|---|---|---|---|---|
| wtPUM | Q1126 | E1083 | Q1047 | Q1013 | Q975 | Q939 | Q903 | Q867 |
| | Y1123 | N1080 | Y1044 | Q1011 | H972 | R936 | Y900 | R864 |
| | N1122 | S1079 | N1043 | C1007 | | C935 | N899 | S863 |
| RNA (5' - 3') | U | G | U | A | A/U/C | A | U | A |

| Repeats | R8 | R7 | R6 | R5 | R4 | R3 | R2 | R1 |
|---|---|---|---|---|---|---|---|---|
| mPUMt | Q1126 | Q1083 | Q1047 | E1013 | S975 | E939 | Q903 | Q867 |
| | Y1123 | N1080 | Y1044 | Q1011 | H972 | R936 | Y900 | R864 |
| | N1122 | N1079 | C1043 | S1007 | N971 | S935 | N899 | N863 |
| RNA (5' - 3') | U | U | A | G | G | G | U | U |

**Supplementary Table 1.** Amino acid mutations used to generate mPUMt.

Target RNA sequences of wild-type PUM-HD (wtPUM) and mutant PUM-HD (mPUMt) are shown at the bottom for each type. mPUMt was generated by inserting 10 amino acid mutations (S863N, C935S, Q939E, 971N, Q975S, C1007S, Q1031E, N1043C, S1079N, and E1083Q). Mutated amino acids and their recognition bases in the PUM-HD repeats are shown, respectively, in blue and red.

| | | Sequence (5'-3') |
|---|---|---|
| TERRA-specific RT-pimer | Reverse | CCCTAACCCTAACCCTAACCCTAACCCTAA |
| GAPDH-specific RT-primer | Reverse | GCCCAATACGACCAAATCC |
| GAPDH | Forward | GCACCGTCAAGGCTGAGAAC |
| | Reverse | TGGTGAAGACGCCAGTGGA |
| 2q TERRA | Forward | AAAGCGGGAAACGAAAAGC |
| | Reverse | GCCTTGCCTTGGGAGAATCT |
| 9p TERRA | Forward | GAGATTCTCCCAAGGCAAGG |
| | Reverse | ACATGAGGAATGTGGGTGTTAT |
| 17p1 TERRA | Forward | CTTATCCACTTCTGTCCCAAGG |
| | Reverse | CCCAAAGTACACAAAGCAATCC |

**Supplementary Table 2.** list of primers used in reverse transcription (RT) and quantitative PCR (qPCR).

**Video 1.** Spatiotemporal dynamics of TERRA in the nucleus.

Time-lapse video of the U2OS cells expressing the TERRA probe and iRFP-TRF1. TERRA molecules were detected as individual fluorescent particles in the nucleus. In the raw images, background fluorescence from the out of focus area was also detected: frame rate, 100 ms/frame; scale, 4 μm.

**Video 2.** Spatiotemporal dynamics of telomere in the nucleus.

Time-lapse video of the U2OS cells expressing TERRA probe and iRFP-TRF1. iRFP-TRF1 represents telomere positions, and was observed as punctate fluorescent spots in the nucleus: frame rate, 100 ms/frame; scale, 4 μm.

**Video 3.** Motions of TERRA and telomere spots reconstructed as Gaussian spots.

TERRA spots and telomere spots from the Video S1 and S2 were fitted, respectively, with Gaussian spots of 200 nm diameter (TERRA, green spot) and 500 nm diameter (telomere, magenta spot): frame rate, 100 ms/frame; scale, 4 μm.

**Video 4.** Stationary TERRA and free-diffusing TERRA in the nucleus

TERRA spots of which diffusion coefficients were lower than those of chemically fixed spots were defined as stationary TERRA (orange spots), whereas the remainder were regarded as free-diffusing TERRA (cyan). The TERRA spots were overlaid with telomeres (magenta): frame rate, 100 ms/frame; scale, 4 μm.

**Video 5.** Formation of a TERRA-hnRNPA1 complex in the nucleus

TERRA and hnRNPA1 were represented in green spots (TERRA probe, EGFP) and red spots (TMR labeled SNAP-hnRNPA1). Overlapping events exceeding 0.2 s (yellow) show a TERRA-hnRNPA1 complex formation: frame rate, 100 ms/frame; scale, 0.5 μm.
